# CsrA coordinates the expression of ribosome hibernation and anti-σ factor proteins

**DOI:** 10.1128/mbio.02585-23

**Published:** 2023-11-09

**Authors:** Christine Pourciau, Helen Yakhnin, Archanna Pannuri, Mark G. Gorelik, Ying-Jung Lai, Tony Romeo, Paul Babitzke

**Affiliations:** 1 Department of Biochemistry and Molecular Biology, Center for RNA Molecular Biology, The Pennsylvania State University, University Park, Pennsylvania, USA; 2 Microbiology and Cell Science, Institute of Food and Agricultural Sciences, University of Florida, Gainesville, Florida, USA; National Institute of Child Health and Human Development (NICHD), Bethesda, Maryland, USA

**Keywords:** ribosome hibernation, CsrA, translational control, anti-sigma factor, protein-RNA interactions

## Abstract

Bacterial growth rate varies due to changing physiological signals and is fundamentally dependent on protein synthesis. Consequently, cells alter their transcription and translation machinery to optimize the capacity for protein production under varying conditions and growth rates. Our findings demonstrate that the post-transcriptional regulator CsrA in *Escherichia coli* controls the expression of genes that participate in these processes. During exponential growth, CsrA represses the expression of proteins that alter or inhibit RNA polymerase (RNAP) and ribosome activity, including the ribosome hibernation factors RMF, RaiA, YqjD, ElaB, YgaM, and SRA, as well as the anti-σ^70^ factor, Rsd. Upon entry into the stationary phase, RaiA, YqjD, ElaB, and SRA expression was derepressed and that of RMF, YgaM, and Rsd was activated in the presence of CsrA. This pattern of gene expression likely supports global protein expression during active growth and helps limit protein production to a basal level when nutrients are limited. In addition, we identified genes encoding the paralogous C-tail anchored inner membrane proteins YqjD and ElaB as robust, direct targets of CsrA-mediated translational repression. These proteins bind ribosomes and mediate their localization to the inner cell membrane, impacting a variety of processes including protein expression and membrane integrity. Previous studies found that YqjD overexpression inhibits cell growth, suggesting that appropriate regulation of YqjD expression might play a key role in cell viability. CsrA-mediated regulation of *yqjD* and ribosome hibernation factors reveals a new role for CsrA in appropriating cellular resources for optimum growth under varying conditions.

**IMPORTANCE:**

The Csr/Rsm system (carbon storage regulator or repressor of stationary phase metabolites) is a global post-transcriptional regulatory system that coordinates and responds to environmental cues and signals, facilitating the transition between active growth and stationary phase. Another key determinant of bacterial lifestyle decisions is the management of the cellular gene expression machinery. Here, we investigate the connection between these two processes in *Escherichia coli*. Disrupted regulation of the transcription and translation machinery impacts many cellular functions, including gene expression, growth, fitness, and stress resistance. Elucidating the role of the Csr system in controlling the activity of RNAP and ribosomes advances our understanding of mechanisms controlling bacterial growth. A more complete understanding of these processes could lead to the improvement of therapeutic strategies for recalcitrant infections.

## INTRODUCTION

Our prior transcriptomics analyses in *Escherichia coli* revealed that CsrA affects the expression of numerous genes involved in the regulation of growth-phase-specific changes to the transcriptional and translational machinery. RNA-seq, CLIP-seq, ribosome profiling, and pull-down assays identified eight mRNAs involved in these processes as potential targets for CsrA regulation (Table 1) (1, 2). Several of these genes were of particular interest due to their important roles in modulating RNAP activity and ribosome hibernation. Disrupted regulation of many of these potential CsrA targets affects bacterial growth, fitness, persistence, stress resistance, and/or gene expression (3–9).

**TABLE 1:** Effects of CsrA on genes related to the regulation of RNAP and ribosome activity^*a*^

Gene	Protein function	Genes in operon	Evidence for CsrA regulation	Evidence for CsrA binding
*rsd*	Regulator of σ^70^, stationary phase protein	*rsd*	RPF, RNA	
*rmf*	Dimerization of 70S ribosomes to 90S dimers	*rmf*	RPF, RNA	CO, PW
*hpf*	Maturation of 90S–RMF ribosome dimers to translationally silent 100S complexes	*hpf*	RPF	CLIP, CO
*raiA*	Stabilization of 70S ribosomes in an inactive state	*raiA*	RPF, RNA	CO
*sra*	Binds to 70S ribosomes in stationary phase; unknown function	*bfr, sra*	RPF, RNA	
*yqjD*	Localization of 70S and 100S ribosomes to the inner cell membrane	*yqjCDEK*	RPF, RNA	CO
*elaB*	Analogous to YqjD	*elaB*	RPF, RNA	CO
*ygaM*	Analogous to YqjD	*ygaM*	RPF, RNA	

^*a*^ Evidence for CsrA regulation: RPF = ribosome-protected fragments (1), RNA = steady-state RNA abundance (1). Evidence for CsrA Binding: CLIP = CsrA RNA CLIP-seq (1), CO = RNA copurified with CsrA-His6 (2), PW = position wt matrix analysis CsrA target prediction study (10).

One of these potential targets of CsrA is Rsd (Regulator of sigma D), which functions as an anti-sigma factor by binding to RpoD (σ^70^) and preventing its interaction with core RNA polymerase (RNAP) (7, 11–13). Because σ^70^ is the most abundant sigma factor and has the highest affinity for RNAP, Rsd plays an important role in sigma factor competition for RNAP (14, 15). Rsd-mediated sequestration of σ^70^ alters the balance between σ^70^ and alternative sigma factors, allowing RpoS (σ^S^) to better compete for available core RNAP (16). Rsd protein levels increase as cell growth begins to decrease and are approximately twofold higher in the stationary phase than during exponential growth (8, 13). Global gene expression of WT cells compared to Δ*rsd* and Δ*rpoS* mutants revealed that ~75% of genes under σ^S^ control were also affected by loss of Rsd (17), illustrating the potential importance of Rsd in the establishment of stationary phase phenotypes. In addition, RpoS can increase *E. coli* persister cell formation (18), suggesting that its regulation by Rsd might also impact σ^S^-dependent development of persister cells.

The remaining seven potential targets for CsrA explored in this study are thought to participate in the process of ribosome hibernation, whereby ribosomes are inactivated and stored for later use. In *E. coli* and other *Gammaproteobacteria*, ribosome-associated inhibitor A (RaiA) binds to vacant 70S ribosomes and stabilizes them in an inactive state (19, 20). Alternatively, ribosomes in *E. coli* can be inactivated via the formation of 100S dimers through the action of the ribosome modulation factor (RMF) (21–24) and hibernation-promoting factor (HPF) (25–27). In *E. coli*, RMF is sufficient and essential for ribosome dimer formation. RMF joins two 70S ribosomes into an inactive 90S dimer, which is then bound by HPF, thereby forming a stable and inactive 100S dimer. The RMF binding site overlaps the area of interaction between the mRNA Shine-Dalgarno (SD) sequence and the anti-SD sequence of the 16S rRNA, interfering with the formation of the anti-SD/SD helix (28, 29). Similar to RpoS, RMF expression was reported to impact *E. coli* persister cell formation (18). HPF and RaiA share similar structures and have similar binding sites located at the tRNA-mRNA interaction site within the channel between the head and body domains of the 30S ribosomal subunit. Consequently, the binding of HPF and RaiA is mutually exclusive. This observation is supported by the opposing effects of HPF and RaiA on the formation of the ribosome dimer (27, 30). For instance, an *E. coli hpf* mutant is deficient in forming ribosome dimers *in vivo* even in the presence of RMF (30). This deficiency is likely due to the presence of RaiA binding in the same location as HPF, which stabilizes vacant 70S ribosomes and precludes the formation of the unstable 90S dimer (26, 31).

Other potential ribosome hibernation factors in *E. coli* are the C-tail anchored inner membrane proteins YqjD, ElaB, and YgaM. All three proteins mediate the localization of 70S and 100S ribosomes to the inner cell membrane during the stationary phase. In addition, the overexpression of YqjD leads to inhibition of cell growth and was proposed to inhibit ribosome activity by binding to the 30S subunit (3). YqjD is also associated with increased persister cell formation (18). While not much is known about YgaM, ElaB was proposed to protect against oxidative and heat shock stress (32). The function of the stationary phase-induced ribosome-associated protein SRA is even more obscure. SRA is tightly associated with the 30S subunit. Although it does not influence the distribution of ribosomes in their different states (33, 34), the close interaction of SRA with the ribosome in the stationary phase suggests that it may have a regulatory role similar to that of hibernation factors (31).

## RESULTS

### CsrA regulates the expression of genes involved in controlling the activity and specificity of the transcription and translation machinery

Previous transcriptomics studies identified at least eight potential CsrA targets involved in controlling the activity and specificity of RNAP and ribosomes (Table 1) (1, 2, 35). These previous results were validated using β-galactosidase reporter assays of translational fusions containing the promoter(s), leader, and initially translated region of each gene fused in frame to *lacZ*. Because a *csrA* deletion mutation results in severe growth defects and genetic instability (36, 37), our experiments were performed in WT and CsrA-deficient strains carrying a transposon insertion (*csrA::kan*), which expresses a protein retaining ~10% residual RNA-binding activity (38, 39). To further elucidate the mechanism of regulation for these genes, we constructed transcriptional fusions containing the promoter of the gene of interest driving the expression of *lacZ,* and leader fusions containing the 5′ leader and the initial coding region of the gene fused to *lacZ* driven by the constitutive *lacUV5* promoter. The combined results of these reporter fusion assays provide insight into the mechanism of CsrA-dependent regulation of each gene, indicating whether CsrA effects are mediated transcriptionally (suggesting indirect regulation by CsrA) or post-transcriptionally (suggesting direct regulation by CsrA). Based on our results, we identified three potential mechanisms of CsrA-dependent regulation for seven of the eight genes tested: (i) direct post-transcriptional repression during exponential growth, (ii) indirect transcriptional repression during exponential growth, and (iii) indirect transcriptional repression during early exponential growth, followed by indirect activation in stationary phase.

**Fig 1:**
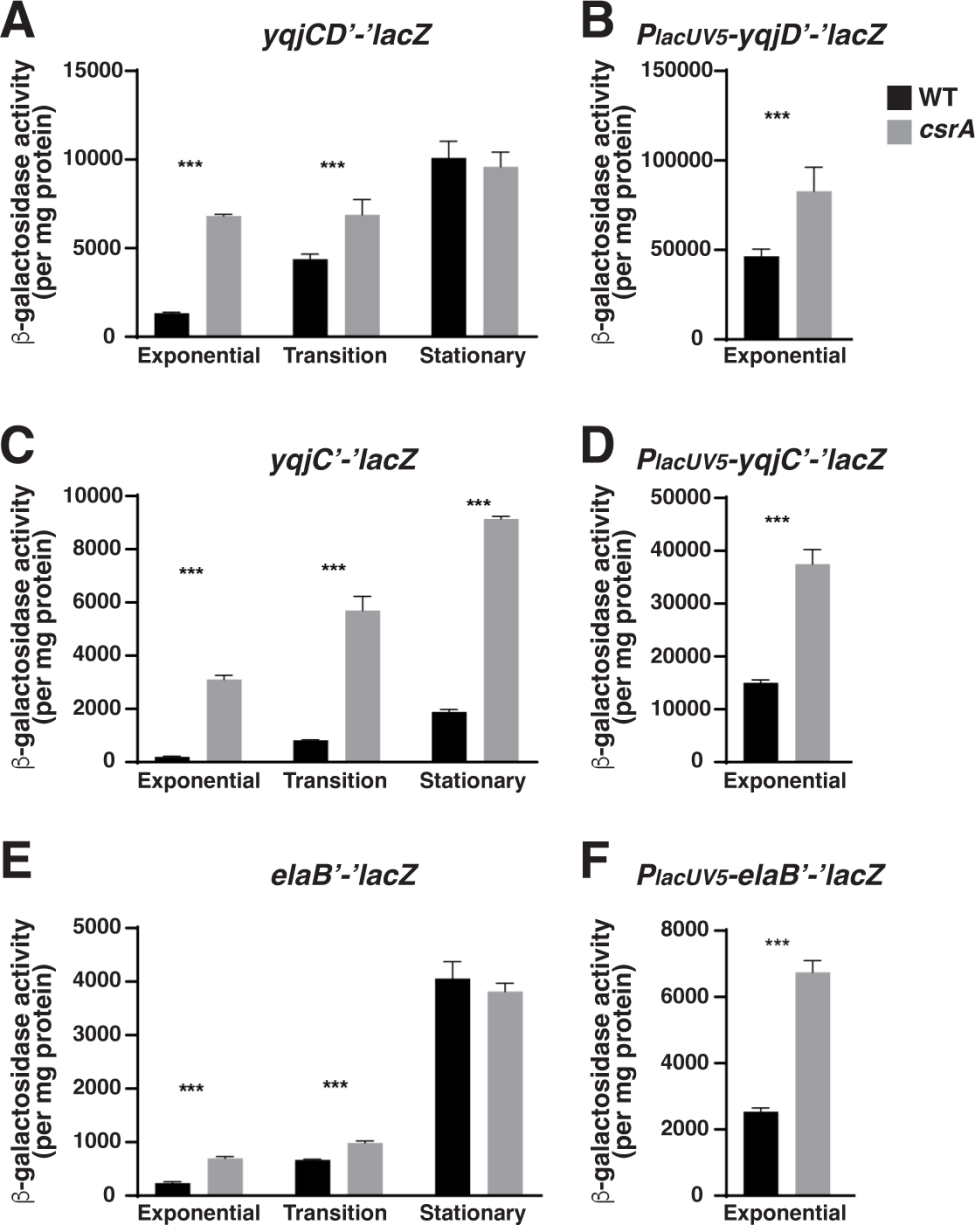
Regulation by CsrA via mechanism 1. Effects of CsrA on the expression of *yqjCD* (**A**), *yqjC* (**C**), and *elaB* (**E**) translational fusions and *yqjD* (**B**), *yqjC* (**D**), and *elaB* (**F**) leader fusions. β-galactosidase activities for all fusions were determined in the exponential phase. β-galactosidase activities were also determined for translational fusions during the transition to the stationary phase and during the stationary phase. Each bar shows the mean and standard deviation from four separate experiments. Statistical significance was determined using unpaired *t*-tests and denoted as follows: ***P < 0.001.

**Fig 2:**
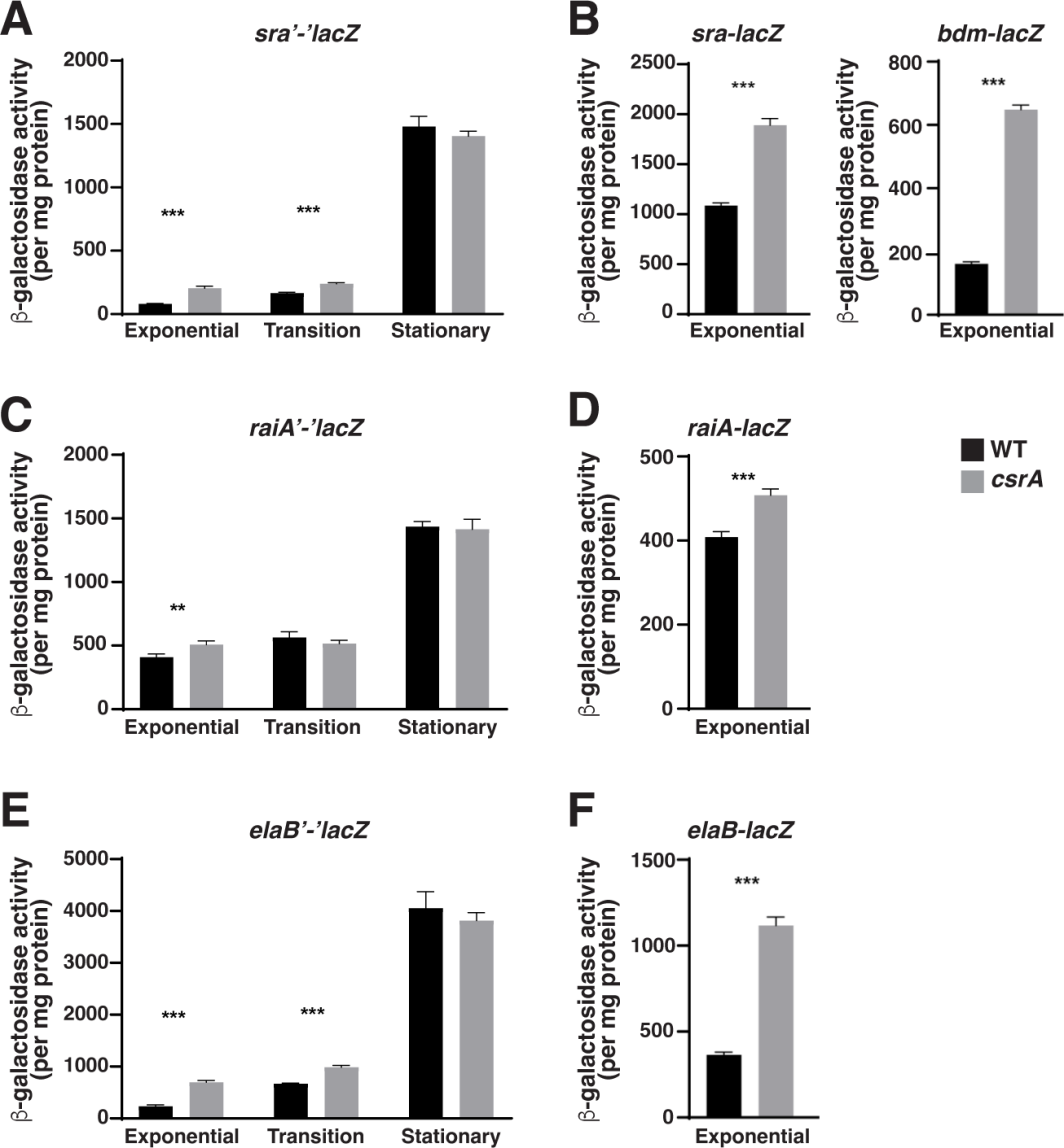
Regulation by CsrA via mechanism 2. Effects of CsrA on the expression of *sra* (**A**), *raiA* (**C**), and *elaB* (**D**) translational fusions and *sra/bdm* (**B**), *raiA* (**D**), and *elaB* (**F**) transcriptional fusions. β-galactosidase activities were determined in the exponential phase. β-galactosidase activities were also determined for translational fusions during the transition to the stationary phase and during the stationary phase. Each bar shows the mean and standard deviation from four separate experiments. Statistical significance was determined using unpaired *t*-tests and denoted as follows: ***P < 0.001 **P < 0.002.

Mechanism 1 genes include the C-tail anchored inner membrane proteins *yqjD* and *elaB* (Fig. 1). Although *yqjD* transcription units are not well defined, computational predictions suggest that it is part of the *yqjCDEK* operon, while RNA-seq enriched for primary transcripts identified a number of putative transcription start sites (TSS) upstream of *yqjD* in the *yqjC* coding region (40). These results suggest that *yqjD* may be co-expressed with *yqjC*, as well as from its own promoter. Consequently, reporter fusions were constructed to measure the effects of CsrA on the expression of transcripts originating from both promoter regions. The *yqjCD'-'lacZ* and *yqjC'-'lacZ* translational fusions were repressed by CsrA during the exponential phase (Fig. 1A and C) and previous transcriptomics revealed that CsrA has strong effects on *yqjC* transcript levels, stability, and ribosome occupancy (1), suggesting that CsrA may regulate this transcript directly. Further support for direct CsrA-mediated regulation of the *yqjCDEK* transcript comes from the *P_lacUV5_-yqjC'-'lacZ* leader fusion, which was repressed by CsrA (Fig. 1D). Notably, the *P_lacUV5_-yqjD'-'lacZ* leader fusion was also repressed (Fig. 1B), indicating that *yqjD* is also expressed and regulated separately from *yqjC*. In contrast to the translational and leader fusions, the expression of a *yqjC-lacZ* transcriptional fusion was not affected by CsrA (data not shown). We infer that CsrA directly regulates *yqjC* and *yqjD* expression post-transcriptionally. Our results further suggest that CsrA regulates *yqjD* expression directly from both the *yqjC* and *yqjD* leaders. Finally, the *elaB* translational (Fig. 1E), leader (Fig. 1F), and transcriptional (Fig. 2F) fusions were all regulated by CsrA during exponential growth, indicating that CsrA regulates *elaB* expression both transcriptionally and post-transcriptionally.

Mechanism 2 genes *sra*, *raiA*, and *elaB* were repressed by CsrA during exponential growth (Fig. 2). *sra* is transcribed from its own promoter, as well as from the upstream *bdm* promoter. Consequently, transcriptional reporters were constructed for both promoters (Fig. 2B). *sra*, *bdm*, and *raiA* reporter expressions were repressed by CsrA in the translational (Fig. 2A, C, and E) and transcriptional (Fig. 2B, D, and F) fusions, but regulation was absent in leader fusions (data not shown). These results indicate that regulation of these genes by CsrA occurs indirectly, presumably via CsrA-dependent regulation of transcription factors.

**Fig 3:**
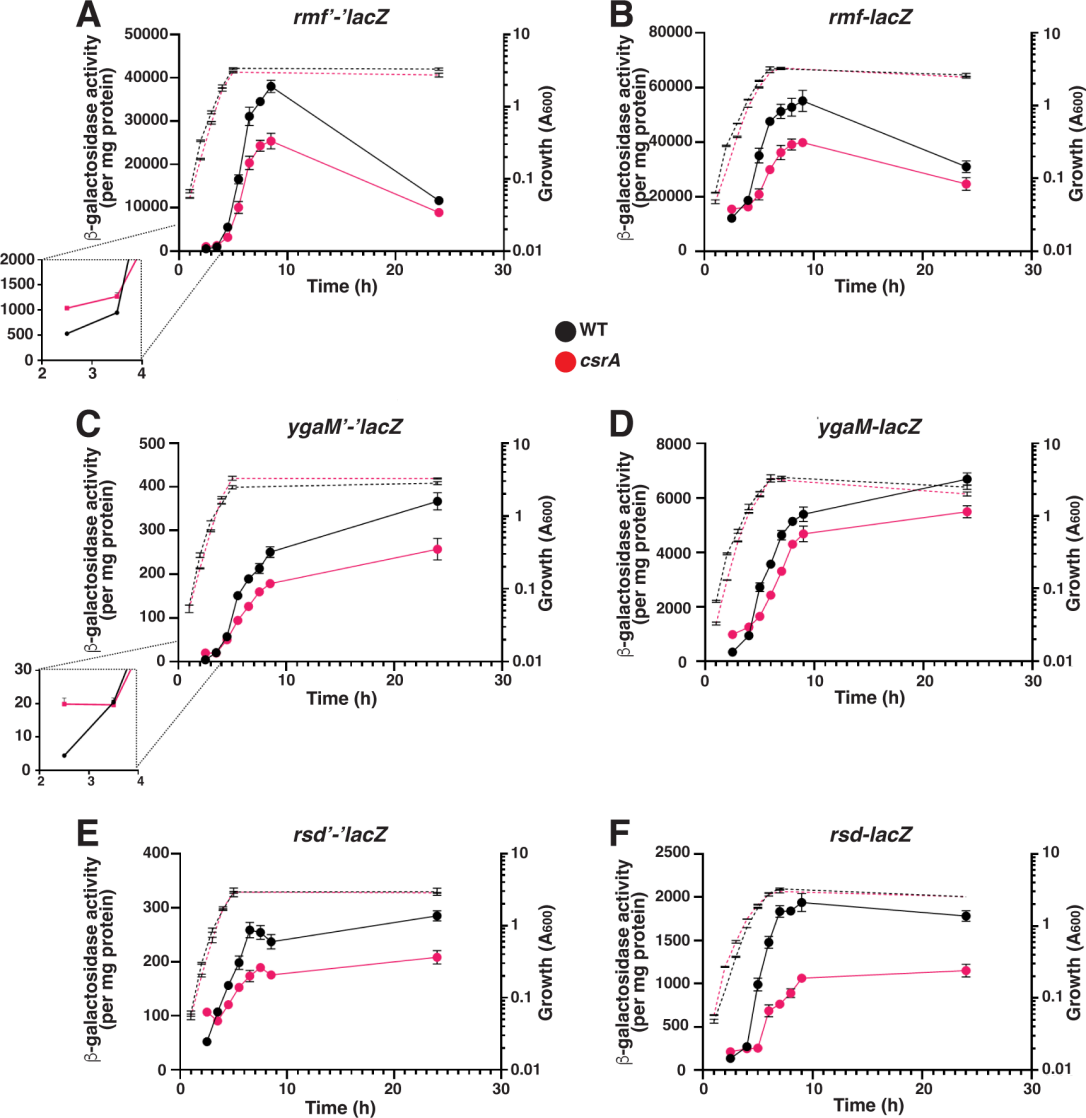
Regulation by CsrA via mechanism 3. Effects of CsrA on the expression of *rmf* (**A**), *ygaM* (**C**), and *rsd* (**E**) translational fusions and *rmf* (**B**), *ygaM* (**D**), and *rsd* (**F**) transcriptional fusions. β-galactosidase activities were determined at eight time points through the exponential, transition to stationary, and stationary phases. Each point shows the mean and standard deviation from four separate experiments.

Mechanism 3 genes are regulated by CsrA in opposing directions during different growth phases and include *rmf*, *ygaM,* and *rsd* (Fig. 3). CsrA repressed *rmf*, *ygaM,* and *rsd* reporter expression during early exponential growth and activated their expression upon entry into stationary phase (Fig. 3A, C, and E). Due to the complicated pattern of CsrA activity, detailed *lacZ* assays were performed to fully assess the role of CsrA in this unusual regulatory pattern. A similar pattern of growth-dependent CsrA regulation was observed for the *rmf, ygaM,* and *rsd* transcriptional fusions (Fig. 3B, D, and F), in which expression was repressed during exponential growth and activated during stationary phase. However, there was no regulation observed for the leader fusions (data not shown), indicating that this regulation occurred transcriptionally. Interestingly, expression of both *rmf* and *rsd* is known to be coordinately regulated by many bifunctional transcription factors that are capable of either activating or repressing transcription (41), so it is possible that the CsrA effects may be mediated via one or more of these shared regulators. Lastly, the expression of *hpf* translational, transcriptional, and leader fusions was not affected by CsrA, indicating that the expression of this gene is not regulated by CsrA (data not shown).

**Fig 4:**
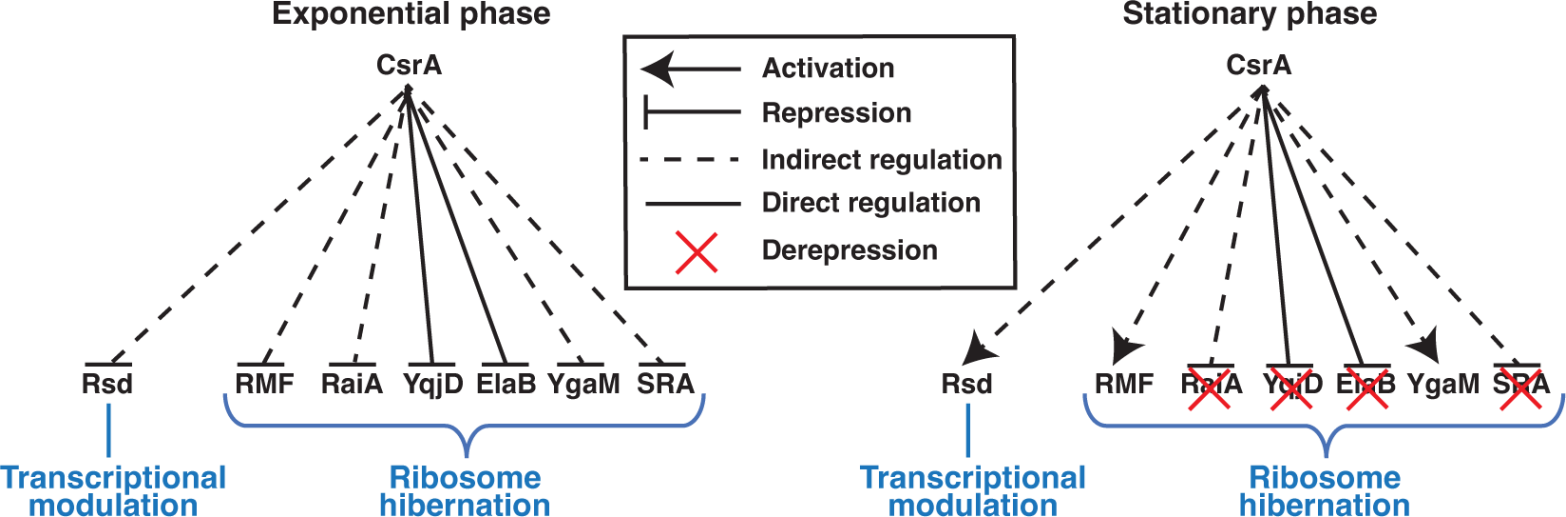
Model for regulating the transcription and translation machinery by CsrA. During exponential growth and in the absence of stress, CsrA represses the expression of *rsd*, *rmf*, *raiA*, *yqjD*, *elaB*, *ygaM*, and *sra*. Presumably, Rsd-mediated sequestration of σ^70^ is repressed and ribosomes are stored and inactivated. Upon induction of stress or stationary-phase growth, CsrB/C sRNAs accumulate and sequester CsrA, which derepresses *raiA*, *yqjD*, *elaB*, and *sra* and activates *rsd*, *rmf*, and *ygaM*, thereby supporting the transcription of stress response genes via σ^S^, and inactivating and sequestering ribosomes to limit global protein synthesis.

To summarize, CsrA regulates seven genes that inhibit the transcription and translation machinery (Fig. 4). During exponential growth, CsrA represses the expression of *rsd*, *rmf*, *raiA*, *yqjD*, *elaB*, *ygaM*, and *sra*. Conversely, during the stationary phase, CsrA activates the expression of *rsd, rmf,* and *ygaM,* and derepresses the expression of *raiA*, *yqjD*, *elaB*, and *sra*. These results implicate the Csr system in growth phase-specific modulation of RNAP and ribosome activity.

### Elucidation of *yqjCDEK* operon structure

Of particular interest to this study was the role of CsrA in regulating the *yqjCDEK* operon. The *yqjC* and *yqjCD* translational fusions exhibited the strongest regulatory effects by CsrA, and regulation of the *yqjC* and *yqjD* leader fusions suggested that the regulation occurred via direct CsrA interaction with the transcripts (Fig. 1). In addition, the overexpression of YqjD resulted in the inhibition of growth (3), indicating that regulation of YqjD levels in the cell may play an important role in maintaining active growth in certain physiological conditions. Similar results were reported for YqjE, with overexpression also resulting in growth arrest (42). Unfortunately, the functions of YqjC, YqjE, and YqjK are unknown, making it difficult to interpret the overall physiological role of the operon. Furthermore, the structure and expression of the *yqjCDEK* operon are complicated and not well understood. As mentioned above, YqjD is likely expressed as part of the *yqjCDEK* operon (40), as well as independently of *yqjC* in one or more alternative transcripts (43). Considerable differences between the expression levels of *yqjC* and *yqjD* support the hypothesis that *yqjD* is expressed from a second promoter within the *yqjC* coding region (40, 44, 45). For example, estimates of protein abundance in *E. coli* MG1655 found that YqjD levels were consistently higher than that of YqjC (up to 20-fold in MOPS medium with glucose) (46). In addition, global TSS mapping using RNA-seq predicted the presence of a TSS upstream of *yqjE* and *yqjK* (47), suggesting that the *yqjCDEK* operon may generate a variety of transcripts containing one or more cistrons.

**Fig 5:**
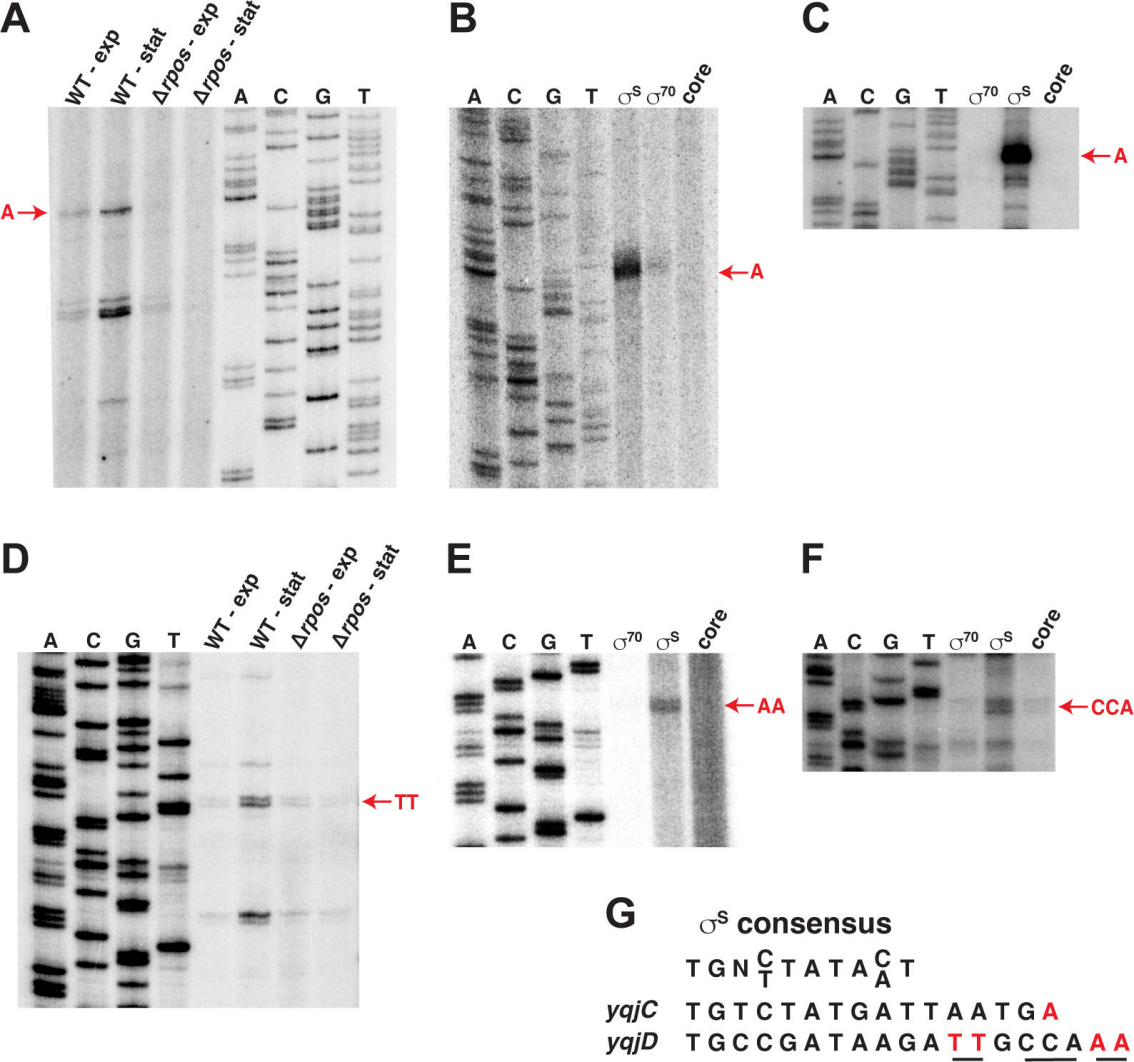
Mapping of the 5′ end of the *yqjC* and *yqjD* transcripts. Primer extension was performed using WT and *rpoS* mutant strains grown in LB. Total RNA was isolated from exponential and early stationary phase cultures. RNA was hybridized to an end-labeled DNA primer and subsequently extended with reverse transcriptase. (**A**) Primer extension products generated from *yqjC* RNA are indicated by PE1-4. (**D**) Primer extension products generated from *yqjD* RNA are indicated by PE1-2. (**B, E**) *In vitro* transcription using reconstituted Eσ^S^, reconstituted Eσ^70^ or core RNA polymerase and *yqjC* (**B**) or *yqjD* (**E**) DNA templates. (**C, F**) Primer extension mapping of the 5′ end of *yqjC* (**C**) or *yqjD* (**F**) transcripts after *in vitro* transcription reactions using reconstituted Eσ^S^, reconstituted Eσ^70^ or core RNA polymerase and only cold UTP. (**G**) The σ^S^ consensus sequence and the predicted *yqjC* and *yqjD* σ^S^ promoters.

To refine our understanding of YqjD expression, primer extension (PE) and *in vitro* transcription assays were performed to identify the TSS of *yqjC* and *yqjD* (Fig. 5). To identify the TSS of *yqjC*, primer extension was performed on total cellular RNA isolated from WT and Δ*rpoS* strains during exponential and stationary phase. In the WT strain, several different PE products were detected weakly in the exponential phase and more intensely in the stationary phase for both *yqjC* and *yqjD*. These products were present at low levels during exponential and stationary growth phase in the Δ*rpoS* strain (Fig. 5A and D), indicative of their expression occurring in a σ^S^-dependent manner. The presence of additional bands may be due to the existence of a strong RNA secondary structure, which can inhibit primer extension. Alternatively, these RNA species could be derived from stable RNA decay intermediates or transcription termination.

Due to the presence of several primer extension products for *yqjC* and *yqjD*, we performed *in vitro* transcription reactions on each gene using Eσ^70^ and Eσ^S^ holoenzymes, as well as core RNA polymerase as a negative control. To identify the authentic *yqjC* TSS and to ascertain the location of the predicted promoter, *in vitro* transcription experiments were performed using a DNA template extending from −255 through +67 relative to the *yqjC* translational start site. To visualize bands on a gel we used [α-^32^P]UTP for body labeling transcripts (Fig. 5B and E) or [γ-^32^P]ATP 5′-labeled primers and reverse transcription of unlabeled transcripts (Fig. 5C and F). The product of the *yqjC in vitro* transcription reaction was a single A (Fig. 5B), which was especially strong in the Eσ^S^ holoenzyme reaction and corresponded to a band in the initial primer extension reaction (Fig. 5A). Thus, we conclude that *yqjC* (and likely also *yqjCDEK*) is expressed from a σ^S^-dependent promoter with a TSS start site at position −82 from the *yqjC* start codon. In addition, it is not uncommon for RNAP driven by σ^70^ to recognize and express genes from σ^S^ promoters, providing an explanation for the low level of transcription of *yqjC in vivo* and *in vitro* by Eσ^70^ (48, 49).

To determine whether *yqjD* is expressed from an additional promoter within the *yqjC* coding region, similar primer extension and *in vitro* transcription assays were implemented (Fig. 5D, E, and F). Subsequent *in vitro* transcription experiments were performed using a DNA template extending from −255 to +11 relative to the *yqjD* translational start site (Fig. 5E). The *in vitro* transcription assay generated a product that did not correspond to any of the PE products, but instead mapped to an AA sequence slightly downstream of one PE product identified in the initial primer extension reaction (Fig. 5E). An additional primer extension reaction performed using the *in vitro*-derived transcript as a template indicated that the 5′ end corresponded to a CCA sequence directly upstream of the AA identified by *in vitro* transcription (Fig. 5F). Although we were unable to precisely define the TSS of the *yqjD* transcript, a σ^S^-dependent promoter is located upstream of the *yqjD* start codon, with a TSS between −105 and −101 relative to the start codon (Fig. 5G). Our results are consistent with a previous study showing that *yqjC*, *yqjD*, and *yqjE* were highly upregulated by RpoS (45), as well as an RNA-seq assay that predicted a TSS for *yqjD* around −109 relative to the start codon (40).

### CsrA directly represses the translation of *elaB*, *yqjC,* and *yqjD*

**Fig 6:**
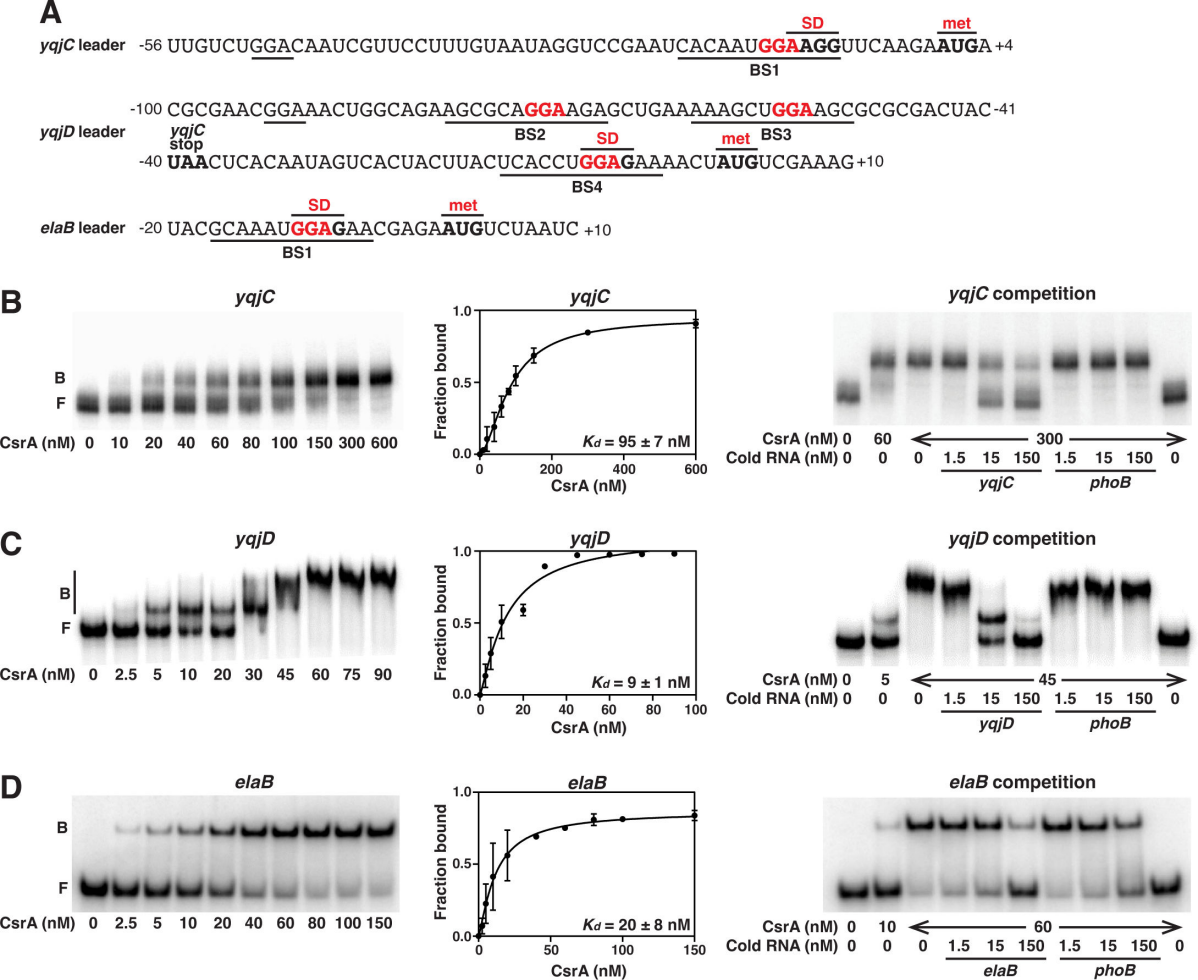
Gel shift analysis of CsrA binding to *yqjC, yqjD,* and *elaB* RNAs. (**A**) Leader sequences with GGA motifs are shown in red. Positions of CsrA binding sites (BS1-4), Shine-Dalgarno (SD) sequences, and translation initiation (met) and stop codons are indicated. GGA sequences that are not part of an authenticated CsrA binding site are underlined. (**B, C, D**) CsrA-RNA gel shift assays, binding curves, and competition assays with *yqjC* (**B**), *yqjD* (**C**), and *elaB* (**D**) transcripts. 5′-end-labeled transcripts (0.1  nM) were incubated with CsrA at the indicated concentrations. Competition reactions were performed in the presence of specific (self) or nonspecific (*phoB*) unlabeled competitor RNAs at the concentrations shown. Images on the left of panels B and D were duplicated in Fig. 8C and A, respectively.

CsrA-dependent regulation of the leader fusions of *yqjD*, *yqjC*, and *elaB* suggested that CsrA interacted with the 5′ leader region of each transcript (Fig. 1B, D, and F). To examine these potential binding interactions, gel shift assays were performed using purified CsrA and *yqjC*, *yqjD*, and *elaB* leader RNAs. CsrA exhibited moderate to high affinity binding to all three RNAs (Fig. 6). A nonlinear least-squares analysis of the data yielded apparent equilibrium binding constant (*K_d_*) values of 95 ± 7  nM, 9 ± 1 nM, and 20 ± 8  nM for the *yqjC*, *yqjD*, and *elaB* RNAs, respectively. These affinities are well within the range of other known mRNA targets of CsrA (50, 51). *yqjC* and *elaB* transcripts revealed a single shifted complex that increased with increasing CsrA concentrations, indicative of a single CsrA dimer bound to each transcript (Fig. 6B and D). However, the *yqjD* transcript displayed a second distinct shifted species at 30  nM CsrA and higher (Fig. 6C), suggesting that two CsrA dimers were bound to those transcripts. Competition assays using specific (self) and nonspecific (*phoB*) unlabeled competitor transcripts confirmed the specific binding of CsrA to each transcript (Fig. 6B, C, and D).

**Fig 7:**
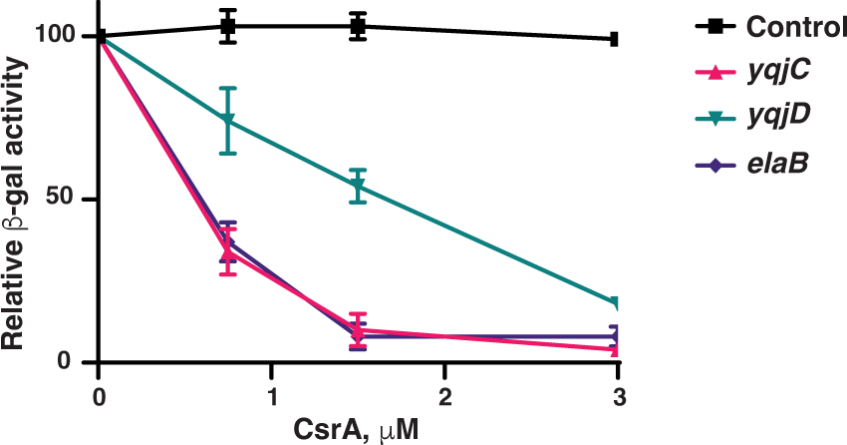
Translation of *yqjC*, *yqjD,* and *elaB* is repressed by CsrA *in vitro*. Coupled transcription-translation reactions were performed with the PURExpress kit using templates containing *yqjC'-'lacZ, yqjD'-'lacZ,* and *elaB'-'lacZ* translation fusions driven by a T7 RNAP promoter. A similar *pnp'-'lacZ* translational fusion was used as a negative control. Purified CsrA was added at the indicated concentrations prior to starting the reaction. Relative β-galactosidase activity depicts the mean ± standard deviation of activity relative to reaction mixtures lacking CsrA. Each point shows the mean and standard deviation from three separate experiments.

Our *in vivo* expression and *in vitro* binding results indicate that CsrA directly regulates *elaB*, *yqjC*, and *yqjD* expression post-transcriptionally (Fig. 1 and 6). To determine whether CsrA represses the translation of these genes, a defined coupled *in vitro* transcription-translation system (PURExpress) was used to measure the expression of leader fusions on plasmid templates. These fusions were essentially identical to those used previously (Fig. 1) except that the *lacUV5* promoter was replaced with a T7 RNAP promoter. Expression of all three fusions was inhibited by CsrA (Fig. 7), whereas a control *pnp*′*-*′*lacZ* fusion was not. We conclude that CsrA represses translation of *elaB*, *yqjC*, and *yqjD* by binding to their 5′ leaders.

### Identification of CsrA binding sites in the *elaB*, *yqjD,* and *yqjC* transcripts

**Fig 8:**
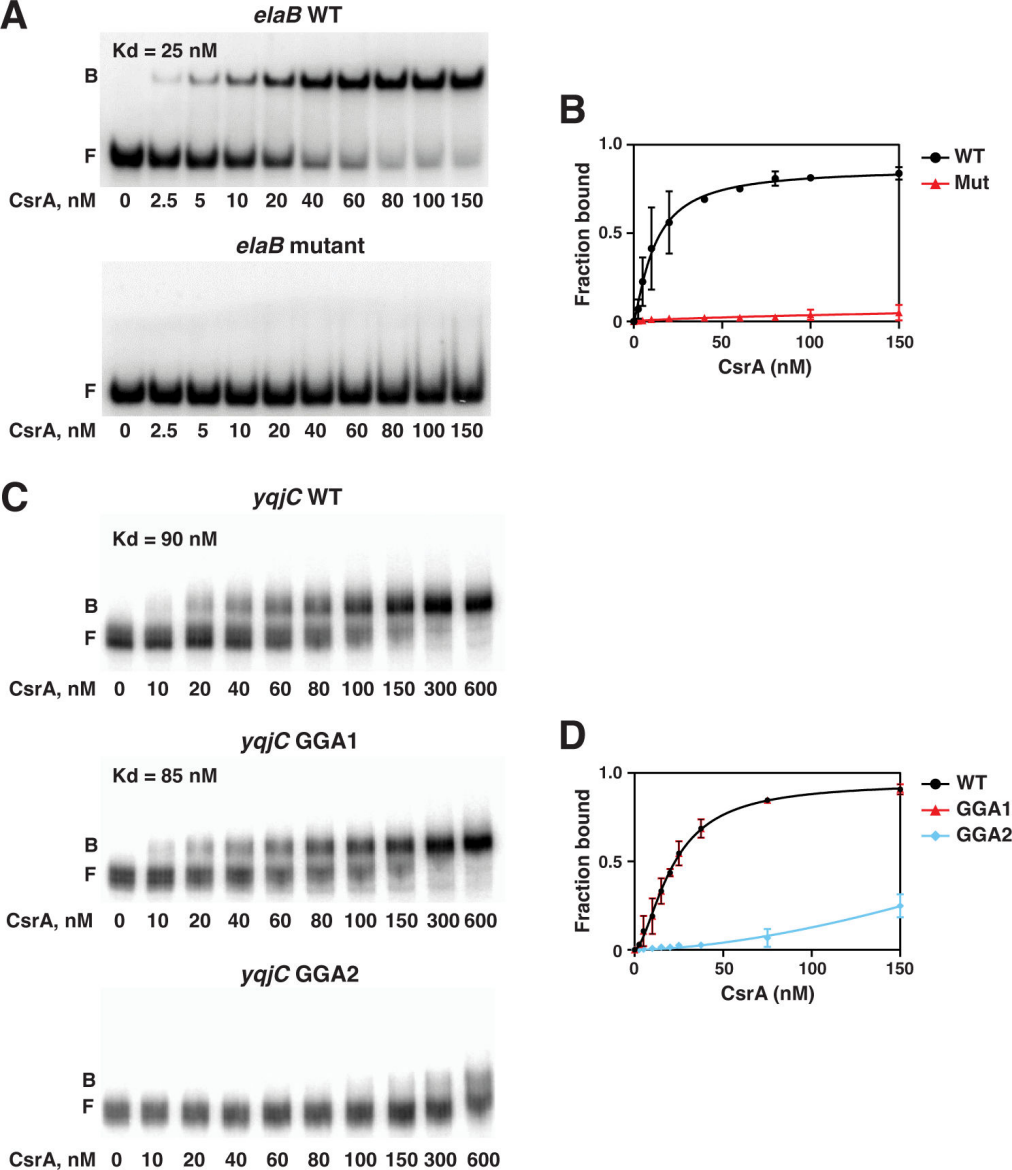
CsrA binding is decreased by mutation of GGA motifs within the *elaB* and *yqjC* leader RNAs. (**A**) Gel shift assays with wild type (WT) and mutant (GGA to CCA) *elaB* RNAs. The *elaB* WT image was duplicated from Fig. 6D. (**B**) Binding curves derived from panel **A**. (**C**) Gel shift assays with WT and mutant (GGA to AAA) *yqjC* RNAs. The *yqjC* WT image was duplicated from Fig. 6B. (**D**) Binding curves derived from panel **C**. 5′-end-labeled transcripts (0.1  nM) were incubated with CsrA at the indicated concentrations.

Putative CsrA binding sites for each transcript were identified based on the presence of conserved GGA motifs, which is a critical component of CsrA binding sites. The *yqjD* RNA that was tested contained four potential CsrA binding sites, while the *yqjC* and *elaB* RNAs contained two and one, respectively (Fig. 6A). The only GGA motif in the *elaB* transcript overlaps the SD sequence (Fig. 6A). To determine whether this sequence is required for CsrA binding, we altered the GGA motif to CCA. mFold predicted that these changes would not affect the transcript’s secondary structure (52). Binding assays using the WT and mutant transcripts revealed that mutating the GGA motif eliminated CsrA binding (Fig. 8A and B), confirming that this sequence is required for CsrA binding and presumably for regulation of *elaB* expression.

Due to the presence of multiple GGA motifs in the *yqjD* and *yqjC* leaders, more extensive analyses were performed to identify CsrA binding sites. The *yqjC* leader is ~80 nucleotides long and contains two potential binding sites, one overlapping the SD sequence (GGA2) and another 34 nt upstream (GGA1) (Fig. 6A). To investigate the role of each GGA motif on CsrA binding to *yqjC* RNA, mutant transcripts were generated that converted the GGA motifs to AAA. Binding assays with the WT, GGA1 mutant, and GGA2 mutant transcripts revealed that disruption of the upstream site (GGA1) had no effect on CsrA binding affinity, whereas altering the downstream GGA2 motif abolished CsrA binding up to a concentration of 600 nM (Fig. 8C and D). We conclude that the GGA motif overlapping the SD sequence is essential for CsrA binding and *yqjC* regulation and will be referred to as binding site one (BS1).

**Fig 9:**
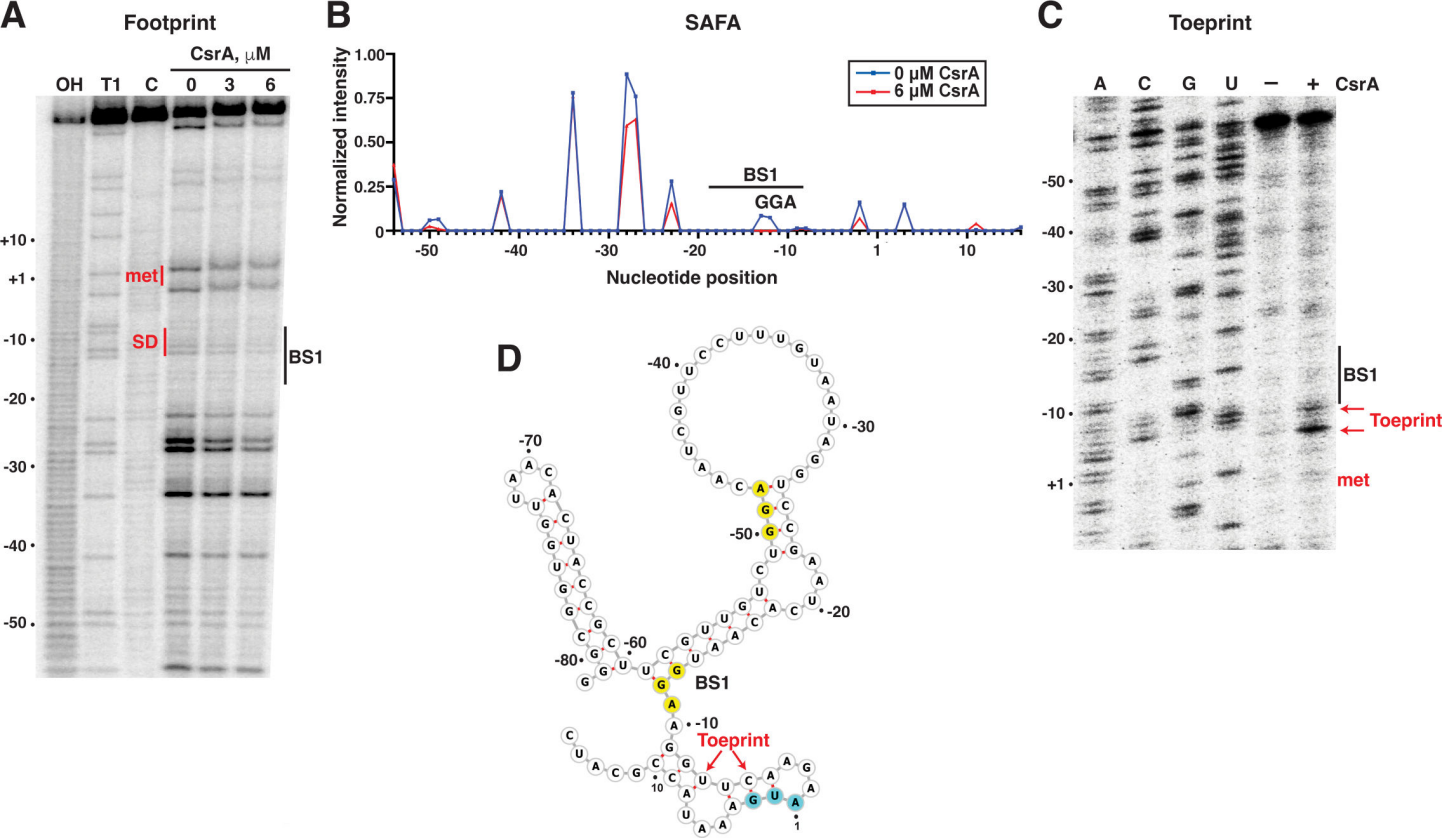
CsrA binds to a site overlapping the *yqjC* SD sequence. (**A**) CsrA-*yqjC* RNA footprint. 5′-end-labeled *yqjC* leader RNA was treated with RNase T1 ± 3 or 6 µM CsrA, as shown. Partial alkaline hydrolysis (OH) and RNase T1 digestion (T1) ladders, as well as a control lane without treatment (**C**), are labeled. Positions of the *yqjC* start codon (met) and the SD sequence overlapping BS1 are shown in red. (**B**) SAFA analysis of CsrA-*yqjC* RNA footprint analysis. The location of the GGA motif within BS1 is indicated. (**C**) CsrA-*yqjC* RNA toeprint using 0.5 U AMV ± 6 µM CsrA. The position of the CsrA toeprints is marked in red. A sequencing ladder is shown. (**D**) Summary of footprint and toeprint results on a structure of the *yqjC* leader predicted by mFold (52) and illustrated in forna (54). BS1 and the toeprint positions are marked. GGA sequences are highlighted in yellow and start codon in cyan. Numbering is with respect to the start of the *yqjC* translation.

To further examine CsrA-*yqjC* RNA interaction, *in vitro* footprinting and toeprinting assays were performed (Fig. 9). CsrA-*yqjC* RNA footprint experiments using RNase T1 as a single-strand G-speciﬁc probe were performed to identify a CsrA binding site(s) in the *yqjC* leader. CsrA preferentially binds to RNAs containing single-stranded GGA motifs, resulting in strong protection from RNase T1-mediated cleavage, making this assay particularly informative. RNase T1 footprinting of the full-length *yqjC* leader RNA showed that CsrA protected GGA2 from RNase T1 cleavage, indicating that CsrA binds to this motif within BS1 (Fig. 9A). Quantitative analysis of these data by semi-automated footprinting analysis (SAFA) (53) confirmed protection of the G residues in BS1 (Fig. 9B). Primer extension inhibition (toeprint) assays were also performed as an alternative method to observe the position of bound CsrA on the *yqjC* transcript. The presence of bound CsrA should stop primer extension by reverse transcriptase, resulting in a toeprint band near the 3′ boundary of the bound protein. Strong toeprint signals were observed at U and C residues a few nts downstream of BS1 (Fig. 9C). We conclude that CsrA binds to BS1 such that bound CsrA blocks ribosome access to the *yqjC* transcript, resulting in translation repression (Fig. 9D).

**Fig 10:**
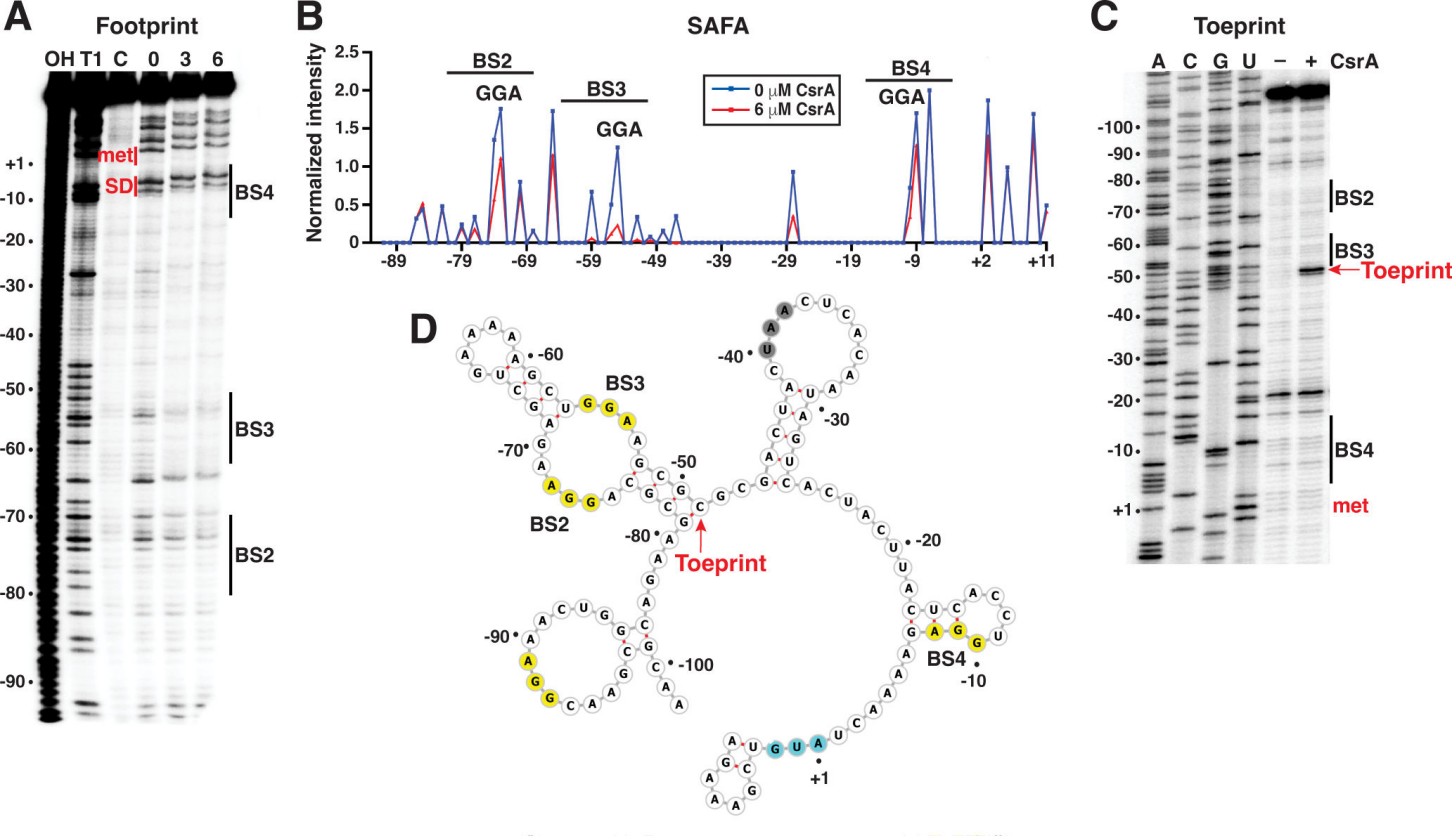
CsrA binds to three sites in the *yqjC-yqjD* intercistronic region. (**A**) CsrA-*yqjD* RNA footprint. 5′-end-labeled *yqjD* RNA was treated with RNase T1 ± 3 or 6 µM CsrA, as shown. Partial alkaline hydrolysis (OH) and RNase T1 digestion (T1) ladders, as well as a control lane without treatment (**C**), are labeled. Positions of the *yqjD* start codon (met) and SD sequence are shown in red. CsrA protected the GGA motifs in BS2, BS3, and BS4 from RNase T1 cleavage. BS1 is found upstream of *yqjD* in the *yqjC* leader. (**B**) SAFA analysis of CsrA-*yqjD* RNA footprint analysis. The locations of three GGA motifs with BS2-4 are indicated. (**C**) CsrA-*yqjD* RNA toeprint using 0.5 U AMV ± 6 µM CsrA. The position of the CsrA toeprint is marked. A sequencing ladder is shown. (**D**) Summary of footprint and toeprint results on a structure of the *yqjD* leader predicted by mFold (52) and illustrated in forna (54). BS2, BS3, and BS4 and the CsrA toeprint is marked. The GGA motifs are highlighted in yellow, the *yqjC* start codon in cyan, and the *yqjC* stop codon in grey. Numbering is with respect to the start of the *yqjD* translation.

As described above, the *yqjC-yqjD* intercistronic region is fairly long and contains four GGA sequences. An mFold prediction of the *yqjD* leader secondary structure indicated the presence of multiple RNA hairpins, including one with an internal loop that contains the first GGA sequence, another with an internal loop that contains the second and third GGAs, while the fourth GGA overlaps the partially paired SD sequence (Fig. 10D) (52). To identify authentic CsrA binding sites in the *yqjD* leader, *in vitro* footprinting and toeprinting assays were performed. RNase T1 footprinting revealed strong CsrA-dependent protection of the second and third GGAs (Fig. 10A). Quantitative analysis of the footprinting data by SAFA (53) confirmed the protection of the G residues surrounding these two GGA motifs (Fig. 10B). Thus, these two regions between −50 and −80 relative to the *yqjD* start codon were designated as the second and third CsrA binding sites (BS2 and BS3) in the *yqjCDEK* operon (BS1 is in the *yqjC* leader). Notably, there was also weak protection of the G residues associated with the fourth GGA overlapping the *yqjD* SD sequence, implying that CsrA also interacts with this region of the transcript (BS4) (Fig. 10A). However, no protection was observed for the first GGA in the *yqjD* leader, indicating that this sequence is not part of an authentic CsrA binding site.

Toeprint assays revealed a strong CsrA-dependent toeprint band just downstream of BS3, providing additional evidence for CsrA binding at this location (Fig. 10C). CsrA-dependent toeprint bands were not observed in the vicinity of BS2 or BS4. The proximity of BS2 to BS3 may have prevented obtaining a toeprint for BS2, whereas CsrA interaction at BS4 may not be strong enough to block reverse transcriptase in this region (Fig. 10C). Taken together, we conclude that CsrA binds to three sites in the *yqjD* leader: BS2, BS3, and BS4 (Fig. 10D). We further infer that binding to BS4 inhibits ribosome binding, leading to repression of *yqjD* translation.

### BS1, BS2, and BS3 are crucial for CsrA-dependent regulation of *yqjC* and *yqjD* expression

**Fig 11:**
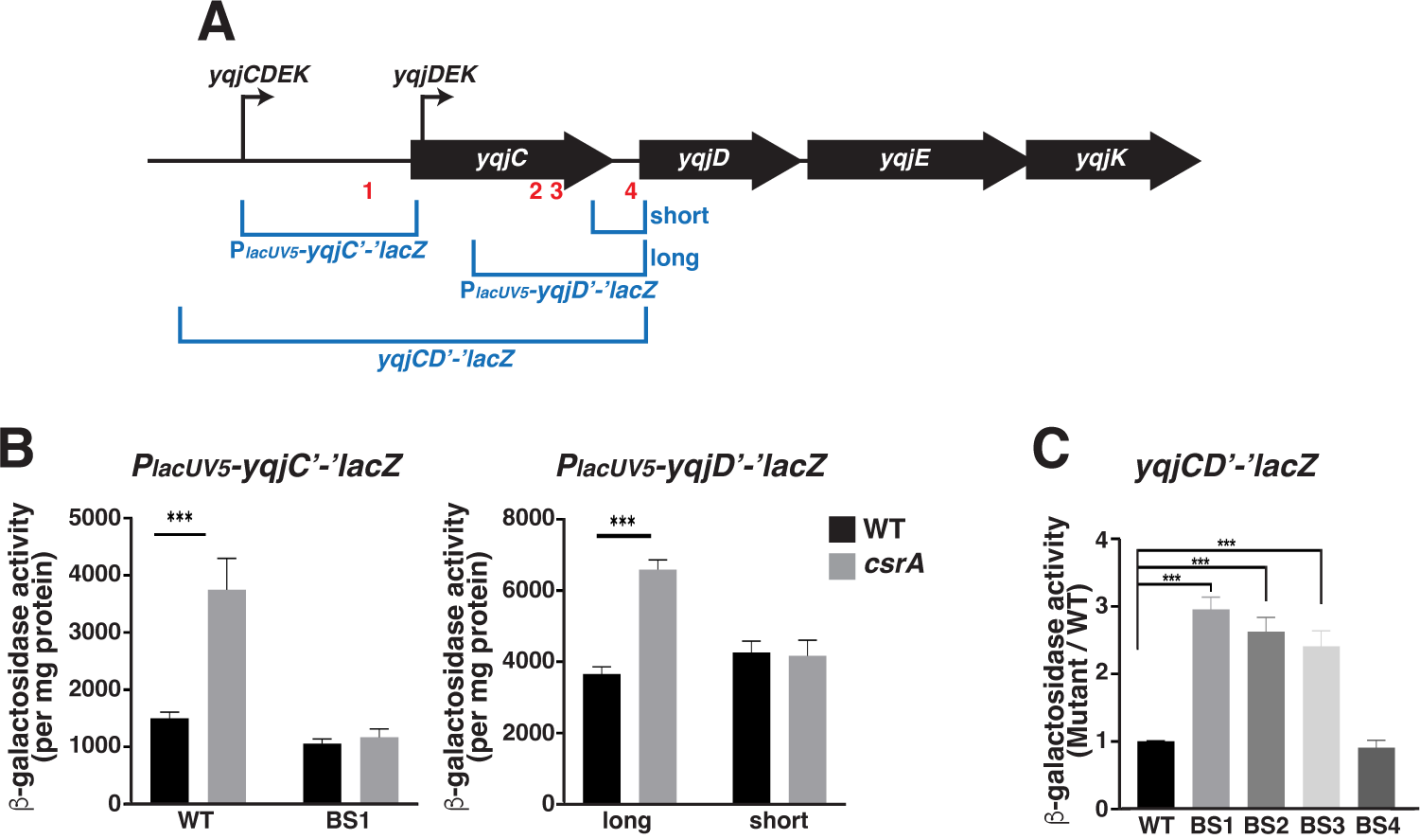
BS1, BS2, and BS3 are crucial for CsrA-dependent regulation of *yqjC* and *yqjD* expression. (A) Diagram of the *yqjCDEK* operon with illustrations of the *P*_*lacUV5*_*-yqjC'-'lacZ, P*_*lacUV5*_*-yqjD'-'lacZ* leader fusions, and the *yqjCD’-‘lacZ* translational fusion in blue. Promoters are indicated with bent arrows, positions of CsrA binding sites are shown in red. Illustration not to scale. (B) Expression of WT and BS1 mutant (AUGGA to GUGGA) *P*_*lacUV5*_*-yqjC'-'lacZ* leader fusions in WT and *csrA* mutant strains (left). Expression of full-length (long) and short *P*_*lacUV5*_*-yqjD'-'lacZ* leader fusions in WT and *csrA* mutant strains (right). (C) Expression of WT and BS1, BS2, BS3, and BS4 mutant *yqjCD'-'lacZ* translational fusions. Results are represented as mutant expression divided by WT expression. (B, C) β-Galactosidase activities per mg of protein ± standard deviations for all reporters were determined in exponential phase (OD_600_ of ~0.5) cultures grown in LB. Each bar shows the mean and standard deviation from four separate experiments. Statistical significance was determined using unpaired *t*-tests and denoted as follows: ***P < 0.001.

To determine the importance of each CsrA binding site on the regulation of the *yqjCDEK* operon, we measured the effect of the binding site (BS1-4) on the expression of chromosomally integrated reporter fusions. To mutate the CsrA binding site within the *yqjC* leader (BS1), we had to avoid disrupting the SD sequence, which is either the GGA motif itself, or AAGG just downstream (the underlined A residue is the same nucleotide). Because of this uncertainty, we introduced an A to G substitution of a residue 2 nt upstream of the GGA motif but still within BS1 (AUGGA to GUGGA) (Fig. 6A). *In vitro* gel shift assays confirmed that this single nucleotide change, A(−15)G, interfered with CsrA binding, resulting in 17-fold lower binding affinity (Fig. S1). Expression of the corresponding WT and mutant *P_lacUV5_-yqjC'-'lacZ* leader fusions was measured in the WT and *csrA::kan* mutant strains during exponential growth. The A(−15)G mutation in BS1 abolished CsrA-dependent regulation, confirming that CsrA binding to this region is necessary for regulation (Fig. 11B). To assess the effect of CsrA on expression of *yqjD* mRNAs that are transcribed from the *yqjDEK* promoter, two similar leader fusions were designed (Fig. 11A). A full-length *P_lacUV5_-yqjD'-'lacZ* fusion included three CsrA binding sites (BS2-4) that are located within and downstream of the *yqjC* coding region, while a short *P_lacUV5_-yqjD'-'lacZ* fusion included the *yqjD* sequence beginning immediately downstream from the *yqjC* stop codon and containing only BS4. While the reporter containing BS2-4 was regulated by CsrA, the short fusion was not (Fig. 11B). These results indicate that the presence of only BS4, which overlaps the *yqjD* SD sequence, is insufficient for CsrA-dependent regulation.

To further characterize the CsrA-dependent regulation of *yqjD* expression, we examined the importance of each individual CsrA binding site on the expression of *yqjCD'-'lacZ* translational fusions. We constructed four translational fusions, each containing one mutated CsrA binding site (BS1-BS4). One fusion contained the previously described A to G mutation in BS1 in the *yqjC* leader. Other fusions contained mutations in BS2 (GGA to GAA), BS3 (GGA to AGA), or BS4 (C to G two nt upstream of the GGA motif to avoid disrupting the overlapping SD sequence) (Fig. 11A). Expression of these fusions was then compared to the WT *yqjCD'-'lacZ* fusion. The mutation in BS1 resulted in threefold higher expression, indicating that CsrA binding upstream of *yqjC* also affects YqjD expression (Fig. 11C). Although this result is somewhat surprising, it could reflect the strong destabilizing effects of CsrA on the *yqjC* transcript observed in the integrated transcriptomics data (1) or possibly the presence of transcriptional polarity triggered by CsrA-mediated repression of *yqjC* translation. In addition, mutations in BS2 and BS3 resulted in increased expression (Fig. 11C), consistent with our *in vitro* results that identified these locations as CsrA binding sites (Fig. 9 and 10). By contrast, the mutation in BS4 had no discernable effect on CsrA-dependent regulation. This result is consistent with BS4 being unable to mediate CsrA-dependent regulation in the absence of BS2 and BS3 (Fig. 11B).

### High-affinity CsrA-*yqjD* RNA interaction requires binding sites 2, 3, and 4

**Fig 12:**
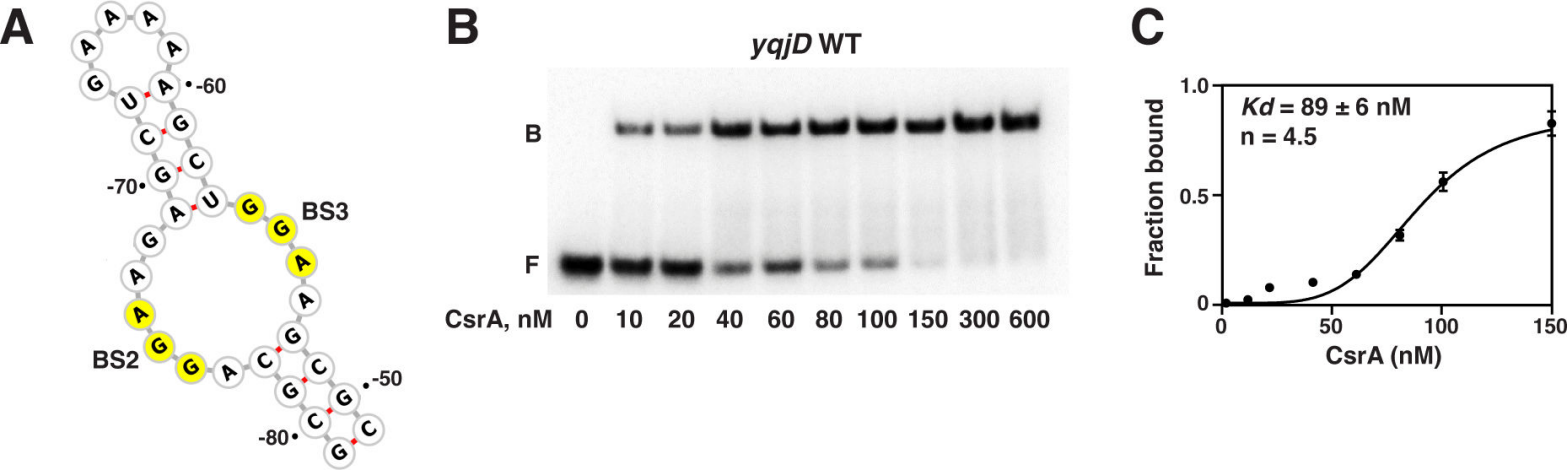
CsrA binds to a *yqjD* transcript containing BS2 and BS3. (**A**) Structure of the RNA fragment predicted by mFold (52) and generated by forna (54). GGA motifs are highlighted in yellow. (**B**) CsrA gel shift assay with the RNA fragment in panel **A**. 5′-end-labeled transcripts (0.1  nM) were incubated with the indicated CsrA concentrations. (**C**) Binding curve derived from panel **B** showing the *K_d_* and Hill coefficient calculated using GraphPad Prism.

To further investigate CsrA-*yqjD* RNA interaction, RNA fragments containing portions of the *yqjD* leader region were examined in gel shift assays with CsrA. A fragment comprised of the predicted RNA structure containing BS2 and BS3 was generated (Fig. 12A), along with mutant forms in which either BS2 (GGA to AAA) or BS3 (GGA to AAA) was mutated (Fig. S3). In addition, a fragment containing only BS4 was generated (Fig. S2A). Mfold predictions of the secondary structure indicated that all fragments, including the ones containing mutations, would fold similarly to the relevant portions of the full-length *yqjD* leader (52). The RNA fragment containing WT BS2 and BS3 exhibited a *K_d_* of 89 ± 6 nM, with a Hill coefficient of 4.5 (Fig. 12B and C), indicating that the binding of CsrA to these two sites is cooperative. Unfortunately, this assay does not permit us to determine whether there are 1 or 2 CsrA dimers bound per RNA molecule. Interestingly, mutations in either binding site virtually eliminated CsrA binding (Fig. S3). In addition, we found that CsrA was unable to bind to an RNA fragment that only contained BS4 (Fig. S2A). These findings are consistent with the *yqjD* expression results demonstrating the importance of BS2 and BS3 for CsrA-dependent regulation, as well as the lack of regulation in the presence of only BS4 (Fig. 11B). However, a full-length *yqjD* transcript with a mutated BS4 exhibited a threefold decrease in affinity compared to the WT transcript, indicating that CsrA binds to BS4 in the presence of BS2 and BS3 (Fig. S2B).

## DISCUSSION

We found that CsrA regulates at least seven genes that modulate the transcription and translation machinery in *E. coli*. Altered expression of many of these genes affects bacterial growth, fitness, stress resistance, and global gene expression (3, 9, 17, 18, 31, 32, 55–59). CsrA-mediated regulation of YqjD and ElaB most likely results in the greatest physiological effects due to the magnitude of CsrA regulation and their critical function(s) in the cell. YqjD and ElaB are C-terminal tail-anchored inner membrane proteins that bind to ribosomes and localize them to the cell membrane. CsrA had the largest effect on the expression of the *yqjCDEK* operon (Fig. 1A and C), with *yqjC* and *yqjD* being repressed approximately 16-fold and 5-fold during exponential growth, respectively. In addition, the expression of *elaB* and *ygaM* was repressed about 3-fold and 4.5-fold, respectively (Fig. 1E and 3C). Despite the regulation observed for *ygaM*, this gene was not investigated further due to the likelihood of it being an indirect CsrA target.

Our results are consistent with a model in which CsrA represses the expression of *rsd*, *rmf*, *raiA*, *yqjD*, *elaB*, *ygaM*, and *sra* in the absence of stress during exponential growth (Fig. 4). CsrA-dependent repression of Rsd relieves σ^70^ sequestration, permitting σ^70^-mediated expression of genes that support active growth (17). CsrA also inhibits the inactivation of ribosomes during active growth by repressing RMF, RaiA, YqjD, ElaB, YgaM, and SRA, an important function given that the active ribosome concentration is rate limiting for growth (60). Upon induction of stress or entry into stationary phase, CsrB/C sRNAs accumulate and sequester CsrA (61), which may result in the observed derepression of RaiA, YqjD, ElaB, and SRA and the indirect activation of RMF, YgaM, and Rsd. Activation of Rsd by CsrA likely supports σ^S^-dependent transcription of stress response genes that are needed during the stationary phase (7, 9). The derepression of RaiA, YqjD, ElaB, and SRA and the activation of RMF and YgaM promote inactivation and sequestration of ribosomes, protecting them from harsh environmental conditions (57, 62–64) and reducing the number of translation-competent ribosomes (27, 65). Thus, CsrA-dependent modulation of hibernation factors helps to maintain the proper balance of global protein synthesis, thereby increasing cellular fitness under a wide variety of physiological conditions (31).

Our finding that CsrA represses *yqjD* translation may be particularly important, although this result of our study is not fully understood. The N-terminal region of YqjD associates with the 30S subunit in 70S and 100S ribosomes and is localized to the cell membrane via a transmembrane motif in its C-terminal region. *E. coli* strains that overexpress YqjD, which occurs to some extent in CsrA-deficient strains, undergo growth arrest (3). This phenotype was also observed in cells that overexpress YqjE (42), a gene that is co-expressed with *yqjD*. Although the functions of YqjC, YqjE, and YqjK are unknown, YqjC is predicted to be a periplasmic protein with a high number of proteoforms (66, 67), while YqjE and YqjK are inner membrane proteins that are predicted to form complexes with themselves and each other (68).

It is well established that RNA structural features in 5′ leader regions and RNA binding proteins that bind to these features can regulate translation initiation (69, 70). In this case, the mechanism of CsrA-mediated regulation of *elaB* and *yqjCDEK* translation is relatively easy to interpret, given the requirement of the 5′ leaders for regulation *in vivo* (Fig. 1), *in vitro* (Fig. 7), and the presence of confirmed CsrA binding sites that overlap the cognate SD sequences (Fig. 9 and 10). The location of the binding sites implies that bound CsrA inhibits translation by blocking ribosome access to the ribosome binding site, a common mechanism of CsrA-dependent regulation (71–75). Because translating ribosomes can protect the transcript from degradation, translational repression often leads to decreased mRNA stability, further reducing protein synthesis. Consequently, this regulatory mechanism results in the observed decreased stability of the *yqjC* transcript (1) and likely contributes to the overall reduction of YqjC and YqjD levels (Fig. 3 to 1A and C).

Furthermore, *yqjD* and its operons *yqjCDEK* and *yqjDEK* are noteworthy for the complex arrangement of CsrA-dependent regulation. In addition to CsrA-dependent repression of YqjD synthesis through its interaction with the upstream *yqjC* leader (BS1), the *yqjD* leader has three CsrA binding sites (BS2-4) that together mediate a fivefold repression of YqjD expression. However, the presence of BS4 alone, which overlaps the *yqjD* SD sequence, is not sufficient for CsrA-mediated regulation of *yqjD* (Fig. 11B). Gel shift assays indicate that CsrA does not bind to BS4 in the absence of BS2 and BS3 (Fig. S2A), and reporter assays show that CsrA-dependent regulation of *yqjD* requires their presence as well (Fig. 11B), indicating that BS2 and BS3 are necessary for full regulation of *yqjD*. Furthermore, a full-length *yqjD* transcript with a mutated BS4 exhibited a threefold decrease in affinity compared to the WT transcript, indicating that CsrA binds to BS4 in the presence of BS2 and BS3 (Fig. S2B). In addition, the observed protection of the G residues associated with BS4, the *yqjD* SD sequence, and the start codon, demonstrate that CsrA interacts with this region of the transcript (Fig. 10A) and thus could inhibit ribosome binding.

Accordingly, we propose a model in which the presence of upstream binding sites is required for a CsrA dimer to stably interact with the low-affinity BS4, leading to translation repression. Interestingly, the presence of both BS2 and BS3 is required for CsrA binding (Fig. S3) and full YqjD regulation (Fig. 11D). The binding of a CsrA dimer to one site results in the cooperative binding of a second CsrA dimer (Fig. 12C). Our best interpretation is that binding of CsrA to BS2 results in “opening” of the RNA secondary structure containing both binding sites, leading to rapid binding of another CsrA molecule to BS3. Previous studies have shown that a CsrA dimer is capable of bridging two sites on a single RNA that are separated by 10 to 63 nt, with optimal spacing >18 nt (71). However, folding of the transcript is likely to impact the distance a CsrA dimer can span. In the *yqjD* transcript, the GGA motifs in BS2 and BS3 are separated from BS4 by 43 and 61 nt, respectively. Consequently, the interaction between BS2 or BS3 and one of the RNA-binding surfaces of a CsrA dimer would allow for the local concentration of CsrA in the vicinity of BS4 to be extremely high. This arrangement would permit rapid binding of the second RNA-binding surface of the CsrA dimer to the low-affinity BS4, which overlaps the SD sequence, resulting in translational repression.

Interestingly, CsrA exhibits different magnitudes and patterns of regulation of YqjC and YqjD. For instance, YqjD is repressed fivefold only during exponential growth, while YqjC is repressed strongly throughout growth, ranging from 16-fold during exponential growth to 5-fold during the stationary phase (Fig. 1). Presumably, the complicated nature of the regulatory system allows CsrA to fine-tune the expression of individual components of the *yqjCDEK* operon. Complicated regulatory pathways are often representative of systems that maintain homeostasis, with complex regulatory circuitry that tightly controls the levels and/or activity of each component. For instance, despite being coexpressed, *yqjC* and *yqjD* seem to respond in opposing ways to environmental conditions. YqjD levels are generally higher than YqjC, especially in minimal media conditions, where YqjD levels increase and YqjC levels decrease (40). In addition, *yqjC* expression is activated and repressed in response to cold stress and oxidative stress, respectively, while *yqjDEK* exhibits the opposite pattern (44). As such, we infer that there is a physiological advantage to the multifaceted regulation of this complex operon.

In conclusion, alterations to gene expression systems (especially the transcription and translation machinery) are a fundamental part of the cellular response to changing environmental conditions and have important consequences for growth. Regulation of these processes has yet to be fully elucidated and knowledge regarding ribosome hibernation factor expression is particularly limited. Given the importance of antibiotic failure in nosocomial and community-acquired infections, the participation of ribosome hibernation in the formation of antibiotic-tolerant persister cells provides additional impetus for the exploration of the regulatory network underlying this process. A more complete understanding could lead to improved therapeutic strategies for recalcitrant infections.

## MATERIALS AND METHODS

### Bacterial strains and culture conditions

The bacterial strains and plasmids used in this study are listed in Table S1. Shaking bacterial cultures were grown in Luria-Bertani (LB) broth at 37°C unless indicated otherwise. When necessary, the following antibiotics were added to the growth media: ampicillin (100  µg/mL), tetracycline (15  µg/mL), gentamicin (10  µg/mL), kanamycin (50  µg/mL), and chloramphenicol (25  µg/mL). Overnight cultures were routinely used to inoculate LB broth unless indicated otherwise. Transduction with P1*vir* was used to introduce gene deletions and disruptions from *E. coli* donor strains constructed in previous studies (38, 76, 77) and from the Keio library (78).

### Construction of reporter fusions

Chromosomally integrated translational, transcriptional, and leader fusions were constructed using the CRIM system (79), plasmid vectors pLFT, pLFX, placUV5 (2), and integrated at the λ*att* site. Single-copy integrates were confirmed by PCR, as described previously (79). Translational fusions were constructed as follows. About 500 nt of DNA upstream of the promoter region through one or more codons downstream of the translational start site was amplified by PCR. Transcriptional fusions were constructed using PCR-generated fragments extending from the promoter region to the transcriptional start site. The amplified PCR products for the transcriptional and translational fusions were digested with PstI and BamHI and ligated into the same sites of pLFT (translational fusions) or pLFX (transcriptional fusions). All pLFT- and pLFX-derived plasmids were electroporated into DH5α λpir cells. The fusion sequences were verified by DNA sequencing and plasmids were isolated and integrated into the *λatt* site of strain MG1655 Δ*lacZ* using the helper plasmid pFINT (2). Leader fusions were constructed using annealed oligonucleotides or gBlocks (Integrated DNA Technologies, IDT) comprising the 5′ leader of each gene. The resulting dsDNA was digested with EcoRI and BamHI and then ligated into the same sites of plasmid placUV5 downstream of the constitutive *lacUV5* promoter. Ligated placUV5 plasmids were integrated directly into the λ*att* site of strain MG1655 Δ*lacZ* using the helper plasmid pFINT (2). This was done to avoid the accumulation of mutations that occurred when the placUV5 plasmid was maintained in DH5α λpir cells, possibly due to the high levels of reporter expression from the constitutive promoter. The fusion sequences were then PCR amplified and sequenced. Refer to Table S2 for primers, oligonucleotides, and gBlock sequences.

### β-Galactosidase assays

Bacterial cultures containing translational, transcriptional, or leader fusions with *lacZ* were grown to exponential phase in LB at 37°C and then diluted to OD_600_ of 0.01 in fresh LB medium. Cells were then harvested at various time points throughout growth. β-galactosidase activity was determined as described previously (2). Total cellular protein was measured using the bicinchoninic acid (BCA) assay (Pierce Biotechnology) following precipitation with ice-cold 10% trichloroacetic acid (vol/vol). Bovine serum albumin served as the protein standard.

### Primer extension assays

Total RNA was isolated using the RNeasy kit (Qiagen) from exponential and stationary phase cultures of strains CF7789 and PLBS982 (CF7789/*rpoS::tet*) grown in LB medium at 37°C. Ten mg of total RNA was hybridized to 150 nM of a ^32^P-5′ end-labeled DNA oligonucleotide complementary to nt + 48 to +67 (relative to the *yqjC* translation start) or to nt −13 to +11 (relative to the *yqjD* translation start) for 3 min at 80°C. Reaction mixtures (10 µL) containing 2 µL of hybridization mixture, 375 µM of each dNTP, 10 mM dithiothreitol (DTT), 200 µg/mL BSA (Promega), 1X SuperScript III buffer and 5 Units SuperScript III Reverse Transcriptase (Life Technologies) was incubated for 30 min at 55°C. Reactions were terminated by the addition of 10 µL of loading buffer (95% formamide, 20 mM EDTA, 0.025% sodium dodecyl sulfate, 0.025% xylene cyanol, and 0.025% bromophenol blue). Samples were fractionated through standard 6% polyacrylamide sequencing gels and visualized with a phosphorimager. Sequencing reactions were performed using pLFT-yqjCD as templates and the same 5′ end-labeled DNA oligonucleotides using the Sequenase version 2.0 DNA Sequencing Kit (Thermo Fisher Scientific).

### Multi-round *in vitro* transcription

Multi-round *in vitro* transcription assays followed a published procedure (80). A 670 bp DNA template (from −255 relative to *yqjC* translational start to +11 relative to *yqjD* translational start) and 322 bp DNA template (from −255 to +67 relative to *yqjC* translational start) were purified with the QIAquick PCR purification kit (Qiagen). Reaction mixtures (6 µL) contained 20 nM DNA template, 40 mM Tris-HCl, pH 8.0, 4 mM MgCl_2_, 0.1 mM EDTA, 4% trehalose, 5 mM DTT, 100 mM KCl, 10% glycerol, 0.27 mM ATP, 0.07 mM CTP, 0.11 mM GTP, 0.11 mM UTP, 0.36 µL [α-^32^P]UTP (3,000 Ci/mmol), and 45 nM reconstituted Eσ^70^, Eσ^S^ or core RNAP. Reconstitution reactions containing 0.4 µM core RNAP (NEB), 4 µM sigma factor, 40 mM Tris-HCl, pH 8.0, 4 mM MgCl_2_, 10% glycerol, 0.1 mM DTT, 100 mM NaCl, and 20 µg/mL BSA were incubated for 15 min at 30°C. *In vitro* transcription reactions were incubated for 30 min at 30°C and the reactions were stopped by adding an equal volume of loading buffer. Reaction mixtures were heated to 90°C for 3 min before fractionating through standard 6% sequencing gels. Radioactive bands were visualized with a phosphorimager.

Identical transcription reactions were made with unlabeled UTP and digested with Turbo DNAse (Thermo Fisher Scientific) for 15 min at 37°C, followed by phenol/chloroform extraction and ethanol purification. RNA pellets were resuspended in TE and then hybridized to 2 µL (500 nM) ^32^P-5′ end-labeled DNA oligonucleotides complementary to nt + 48 to +67 (relative to *yqjC* translation start) or to nt −13 to +11 (relative to *yqjD* translation start) by incubation for 3 min at 55°C. Following the addition of an 8 µL primer extension mixture, the resulting reaction mixture was incubated for 1 h at 37°C. The primer extension mixture for one reaction contained: 3 µL 5X FS buffer for Superscript III, 1.5 µL 0.1 mM DTT, 1.5 µL 2.5 mM dNTP, 0.4 µL RNasin (Promega), 1.1 µL water, and 0.5 µL Superscript III. Reactions were terminated by the addition of a 10 µL loading buffer. Samples were heated for 2 min at 95°C prior to fractionation through standard 6% polyacrylamide sequencing gels. Radioactive bands were visualized using a phosphorimager.

### Footprint assay

CsrA-RNA footprint assays followed a published procedure (72). *yqjC* RNA (from nt −83 to +40 relative to the *yqjC* translational start) and *yqjD* RNA (nt −111 to +9 relative to the *yqjD* translational start) were synthesized with the RNAMaxx kit (Agilent technologies) using PCR-generated DNA templates. Gel-purified RNA was dephosphorylated and then 5′ end-labeled using T4 polynucleotide kinase and [γ-^32^P]ATP (7,000 Ci/mmol). Labeled RNAs were renatured by heating for 1 min at 90°C followed by 10 min of slow cooling at room temperature. Binding reactions (10 µL) contained 2 nM labeled RNA, 10 mM Tris-HCl, pH 7.5, 10 mM MgCl_2_, 100 mM KCl, 40 ng of yeast RNA, 7.5% glycerol, 1 µg of acetylated BSA, 0.1 mg/mL xylene cyanol, and different concentrations of purified CsrA-His_6_. After a 30-min incubation at 37°C to allow for CsrA-RNA complex formation, RNase T1 (0.016 U) was added, and incubation was continued for 15 min at 37°C. Reactions were stopped by adding 10 µL of loading buffer. Samples were heated for 5 min at 90°C and fractionated through standard 6% sequencing gels. Cleavage patterns were examined using a phosphorimager. Quantitative analysis of the CsrA-*yqjD* footprinting results was performed using semi-automated footprinting analysis (SAFA) (53).

### Toeprint assay

CsrA-RNA toeprint assays followed a published procedure (72). Gel-purified *yqjD* RNA (150 nM) in TE buffer was hybridized to a 5′ end-labeled DNA oligonucleotide (150 nM) complementary to the vector-derived 3′ extension by heating for 3 min at 85°C followed by slow cooling to room temperature. Toeprint reaction mixtures (10 µL) contained 2 µL of the hybridization mixture (30 nM final concentration), 1 µM CsrA-His_6_, 375 µM each dNTP, and 10 mM DTT in AMV reverse transcriptase buffer. Mixtures were incubated for 30 min at 37°C to allow CsrA-RNA complex formation. AMV reverse transcriptase (Sigma Aldrich) (0.5–2 U) was added, and incubation was continued for 15 min at 37°C. Reactions were terminated by the addition of 10 µL of gel loading buffer. Samples were heated to 90°C for 5 min and fractionated through standard 6% sequencing gels. Toeprint patterns were visualized with a phosphorimager.

### Gel shift assay

The binding of CsrA to *yqjC*, *yqjD*, and *elaB* transcripts was determined using *in vitro*-synthesized *yqjC*, *yqjD*, and *elaB* transcripts (MEGAshortscript T7 kit; Ambion) and recombinant CsrA-His_6_ (81). DNA templates for *in vitro* transcription containing the T7 RNAP promoter were generated by PCR using plasmid DNA or gBlocks (IDT) as templates. The resulting transcripts were purified via denaturing polyacrylamide gel electrophoresis followed by phenol-chloroform extraction and ethanol precipitation. Transcripts were treated with Antarctic phosphatase (New England Biolabs, NEB) and radiolabeled at the 5′ end using [γ-^32^P]ATP and T4 polynucleotide kinase (NEB). Binding reactions (10 µL) contained 0.1  nM RNA, 10  mM MgCl_2_, 100  mM KCl, 32.5  ng total yeast RNA, 20  mM DTT, 7.5% glycerol, 4 U SUPERasin (Ambion), and various concentrations of recombinant CsrA-His_6_ and were incubated for 30  min at 37°C. Reaction mixtures were separated into 6% native polyacrylamide gels (for *yqjC* mRNA), 9% native polyacrylamide gels (for *yqjD* mRNA), and 12% native polyacrylamide gels (for *elaB* mRNA) with 1 × Tris-borate-EDTA (TBE) buffer. Competition assays were performed in the presence or absence of unlabeled specific (self) and nonspecific (*phoB*) RNA competitors using the minimum CsrA concentration required for a full shift. Labeled RNA was analyzed using a phosphorimager equipped with Quantity One software (Bio-Rad), as previously described (2). The apparent *K_d_* values for CsrA-RNA complex formation were calculated according to a previously described binding equation (82). Gel images from Fig. 6B and D were duplicated in Fig. 8C and A, respectively. In Fig. 6, we compare CsrA binding to three different WT transcripts (*yqjC, ygjD,* and *elaB*), whereas in Fig. 8, we compare the binding of WT and mutant transcripts (*elaB* and *yqjC*).

### Coupled transcription-translation assay

*In vitro* coupled transcription-translation assays followed a published procedure (73). Plasmid pYH411 contains a T7 promoter driving transcription of a *yqjC'-'lacZ* translational fusion containing nt −83 to +5 relative to the *yqjC* start codon. Plasmid pYH412 contains a T7 promoter driving transcription of a *yqjD'-'lacZ* translational fusion from nt −111 to +11 relative to the *yqjC* start codon. Plasmid pYH413 contains a T7 promoter driving transcription of an *elaB'-'lacZ* translational fusion from nt −26 to +2 relative to the *elaB* start codon. Plasmids were used as templates for coupled transcription-translation reactions using the PURExpress kit according to the manufacturer’s instructions (NEB). A similar P_T7_-*pnp'-'lacZ*  translational fusion was used as a negative control. Each 6.7 µL reaction contained 250 ng of plasmid DNA template and various concentrations of purified CsrA-His_6_ with 1 U of RNase inhibitor (Promega) and 2.5 mM DTT, 2.7 µL of solution A and 2 µL of solution B. The mixtures were incubated for 2 h at 37°C and β-galactosidase activity was determined. OD_420_ values without CsrA were normalized to 100%.

### Site-directed mutagenesis

To engineer the mutant reporter fusions for *in vivo* expression assays, site-directed mutagenesis of the *yqjC* and *yqjD* translational reporter plasmids (pCEP125 and pCEP126, respectively) were performed using the QuikChange XL site-directed mutagenesis kit (Agilent) according to the manufacturer’s instructions except for the replacement of the XL10-Gold ultracompetent cells with DH5α λpir competent cells. The pLFT-derived plasmids (pCEP125 and pCEP126) contain a pir-dependent origin of replication and cannot be maintained in the XL10-Gold cells. An A to G substitution 2 nt upstream from the GGA motif in the *yqjC* and *yqjCD* fusions (BS1) was introduced into plasmids carrying the WT reporter fusions. The GGA motif alterations in the *yqjD* reporter fusions were constructed as follows (order based on the *yqjD* TSS). The first GGA motif was changed to AGA, the second to GAA, the third to AGA, and the fourth was altered from G to C 2 nt upstream from the GGA motif. The primer sequences used to introduce the nucleotide changes are found in Table S2. Each fusion was integrated into the AP379 chromosome.

## SUPPLEMENTAL MATERIAL

Fig. S1 mbio.02585-23-s0001.pdf BS1 within the *yqjC* leader is important for CsrA binding.

Fig. S2 mbio.02585-23-s0002.pdf BS4 contributes to CsrA-*yqjD* RNA interaction.

Fig. S3 mbio.02585-23-s0003.pdf BS2 and BS3 are critical for high affinity CsrA-*yqjD* RNA interaction.

Table S1 mbio.02585-23-s0004.docx Strains, plasmids, and bacteriophage used in this study.

Table S2 mbio.02585-23-s0005.docx Primers used in this study.

ASM does not own the copyrights to Supplemental Material that may be linked to, or accessed through, an article. The authors have granted ASM a non-exclusive, world-wide license to publish the Supplemental Material files. Please contact the corresponding author directly for reuse.
