# Supplementary material for: CsrA coordinates the expression of ribosome hibernation and anti-σ factor proteins
Source: mBio. 2023 Nov 9;14(6):e02585-23. doi: 10.1128/mbio.02585-23 (PMC10746276; doi:10.1128/mbio.02585-23)
Supplement: Fig. S1 — BS1 within the yqjC leader is important for CsrA binding. [file mbio.02585-23-s0001.pdf]

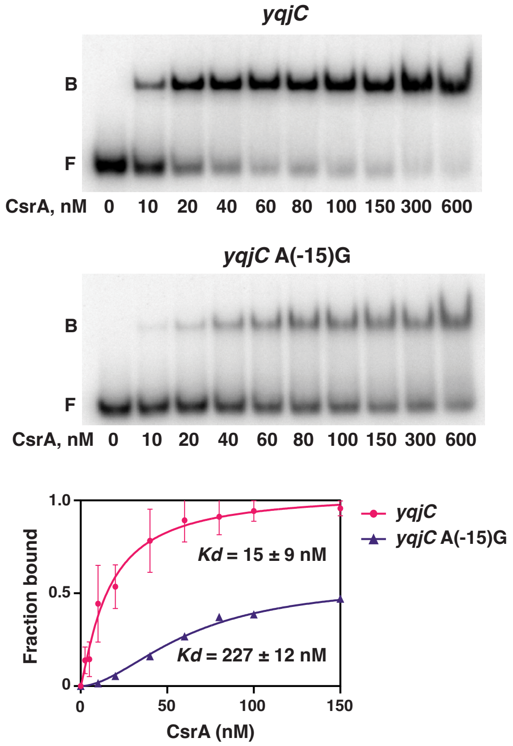

Figure S1. BS1 within the *yqjC* leader is important for CsrA binding.
CsrA-*yqjC* leader RNA binding assay with wild type (top) and mutant (bottom) RNA. The mutation in BS1 [A(-15)G] is 2 nt upstream of the GGA motif. 5′-end-labeled transcripts (0.1 nM) were incubated with indicated CsrA concentrations. Binding curves of CsrA-*yqjC* leader RNA interaction is shown.
