# Supplementary material for: CsrA coordinates the expression of ribosome hibernation and anti-σ factor proteins
Source: mBio. 2023 Nov 9;14(6):e02585-23. doi: 10.1128/mbio.02585-23 (PMC10746276; doi:10.1128/mbio.02585-23)
Supplement: Fig. S2 — BS4 contributes to CsrA-yqjD RNA interaction. [file mbio.02585-23-s0002.pdf]

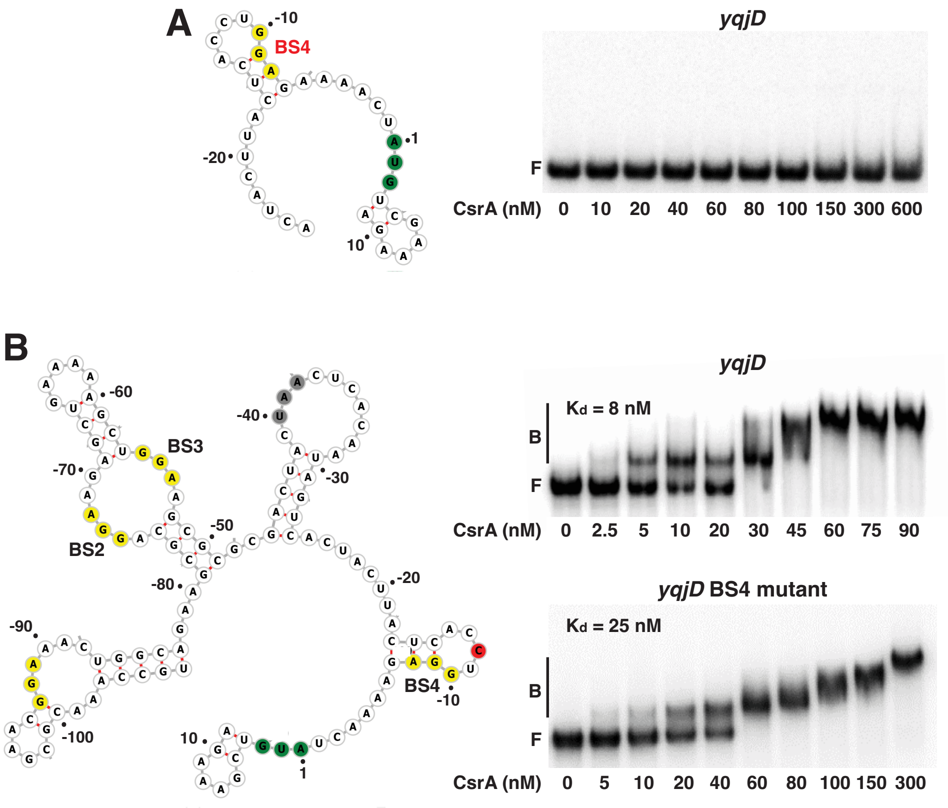

Figure S2. BS4 contributes to CsrA-*yqjD* RNA interaction.
(A) Structure of the BS4 mutant *yqjD* RNA fragment predicted by mFold (50) and generated by *forna* (86). Gel shift assay with the RNA shown at the left. (B) Structure of the full-length *yqjC*-*yqjD* intercistronic RNA. Gel shift assay with WT and BS4 mutant (CUGGA to GUGGA) RNA shown at the right. (A,B) The GGA motifs are highlighted in yellow, start codon in green, with CsrA binding site mutations in red. 5'-end-labeled transcripts (0.1 nM) were incubated with the indicated CsrA concentrations.
