# Supplementary material for: CsrA coordinates the expression of ribosome hibernation and anti-σ factor proteins
Source: mBio. 2023 Nov 9;14(6):e02585-23. doi: 10.1128/mbio.02585-23 (PMC10746276; doi:10.1128/mbio.02585-23)
Supplement: Fig. S3 — BS2 and BS3 are critical for high affinity CsrA-yqjD RNA interaction. [file mbio.02585-23-s0003.pdf]

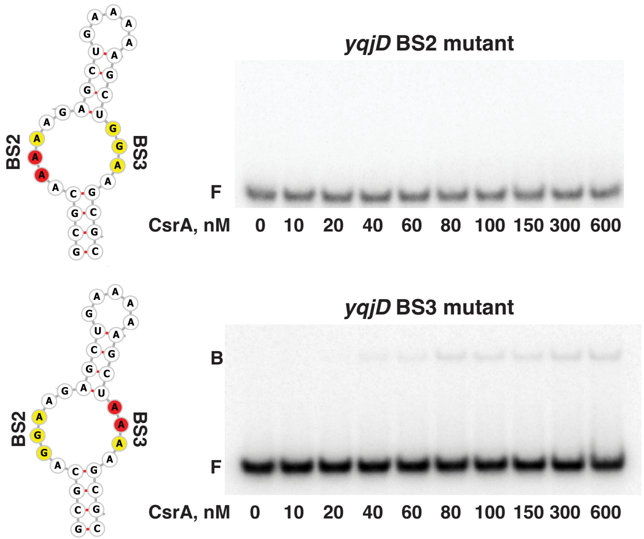

Figure S3. BS2 and BS3 are critical for high affinity CsrA-*yqjD* RNA interaction. Structure of the BS2 and BS3 mutant *yqjD* RNA fragments predicted by mFold (50) and generated by *forna* (86). GGA motifs are highlighted in yellow, with CsrA binding site mutations in red. CsrA-gel shift assays with the indicated mutant transcripts. 5′-end-labeled transcripts (0.1 nM) were incubated with the indicated CsrA concentrations.
