# Supplementary material for: CsrA coordinates the expression of ribosome hibernation and anti-σ factor proteins
Source: mBio. 2023 Nov 9;14(6):e02585-23. doi: 10.1128/mbio.02585-23 (PMC10746276; doi:10.1128/mbio.02585-23)
Supplement: Table S1 — Strains, plasmids, and bacteriophage used in this study. [file mbio.02585-23-s0004.docx]

Table S1. Strains, plasmids, and bacteriophage used in this study

| Name | Genotype or description^a,b^ | Reference or Source |
| --- | --- | --- |
| *E. coli strains* |  |  |
| MG1655 | F^−^ λ^−^ rph-1 | CGSC (no. 6300) |
| AP379 | MG1655 ΔlacZ | (1) |
| TRMG1655 | MG1655 *csrA*::*kan* Km^r^ | (2) |
| CP101 | DH5α *λpir*/pLFT*bdm:sra′-′lacZ* Ap^r^ | This study |
| CP103 | DH5α *λpir*/pLFT*elaB′-′lacZ* Ap^r^ | This study |
| CP105 | DH5α *λpir*/pLFT*hpf′-′lacZ* Ap^r^ | This study |
| CP107 | DH5α *λpir*/pLFT*raiA′-′lacZ* Ap^r^ | This study |
| CP109 | DH5α *λpir*/pLFT*rmf′-′lacZ* Ap^r^ | This study |
| CP111 | DH5α *λpir*/pLFT*rsd′-′lacZ* Ap^r^ | This study |
| CP113 | DH5α *λpir*/pLFT*ygaM’-‘lacZ* Ap^r^ | This study |
| CP115 | DH5α *λpir*/pLFT*yqjC’-‘lacZ* Ap^r^ | This study |
| CP117 | DH5α *λpir*/pLFT*yqjCD’-‘lacZ* Ap^r^ | This study |
| CP119 | DH5α *λpir*/pLFX*bdm-lacZ* Ap^r^ | This study |
| CP121 | DH5α *λpir*pLFX*elaB-lacZ* Ap^r^ | This study |
| CP123 | DH5α *λpir*/pLFX*hpf-lacZ* Ap^r^ | This study |
| CP125 | DH5α *λpir*/pLFX*raiA-lacZ* Ap^r^ | This study |
| CP127 | DH5α *λpir*/pLFX*rmf-lacZ* Ap^r^ | This study |
| CP129 | DH5α *λpir*/pLFX*rsd*1*-lacZ* Ap^r^ | This study |
| CP131 | DH5α *λpir*/pLFX*rsd2-lacZ* Ap^r^ | This study |
| CP133 | DH5α *λpir*/pLFX*sra-lacZ* Ap^r^ | This study |
| CP135 | DH5α *λpir*/pLFX*ygaM-lacZ* Ap^r^ | This study |
| CP137 | DH5α *λpir*/pLFX*yqjC-lacZ* Ap^r^ | This study |
| CP139 | DH5α *λpir*/pLFX*yqjD-lacZ* Ap^r^ | This study |
| CP141 | DH5α *λpir*/placUV5*bdm′-′lacZ* Ap^r^ | This study |
| CP143 | DH5α *λpir*/placUV5*elaB′-′lacZ* Ap^r^ | This study |
| CP145 | DH5α *λpir*/placUV5*hpf′-′lacZ* Ap^r^ | This study |
| CP147 | DH5α *λpir*/placUV5*raiA′-′lacZ* Ap^r^ | This study |
| CP149 | DH5α *λpir*/placUV5*rmf′-′lacZ* Ap^r^ | This study |
| CP151 | DH5α *λpir*/placUV5*rmf′-′lacZ* Ap^r^ | This study |
| CP153 | DH5α *λpir*/placUV5*rsd′-′lacZ* Ap^r^ | This study |
| CP155 | DH5α *λpir*/placUV5*sra′-′lacZ* Ap^r^ | This study |
| CP157 | DH5α *λpir*/placUV5*ygaM′-′lacZ* Ap^r^ | This study |
| CP159 | DH5α *λpir*/placUV5*yqjC*’*-‘lacZ* Ap^r^ | This study |
| CP161 | DH5α *λpir*/placUV5*yqjCD*’*-‘lacZ* Ap^r^ | This study |
| CP163 | AP379/integrated pLFT*bdm:sra′-′lacZ* (-814 to +100) Ap^r^ | This study |
| CP165 | AP379/integrated pLFT*elaB′-′lacZ* (-500 to +31) Ap^r^ | This study |
| CP167 | AP379/integrated pLFT*hpf′-′lacZ* (-641 to +277) Ap^r^ | This study |
| CP169 | AP379/integrated pLFT*raiA′-′lacZ* (-501 to +55) Ap^r^ | This study |
| CP171 | AP379/integrated pLFT*rmf′-′lacZ* (-489 to +78) Ap^r^ | This study |
| CP173 | AP379/integrated pLFT*rsd′-′lacZ* (-497 to +157) Ap^r^ | This study |
| CP175 | AP379/integrated pLFT*ygaM’-‘lacZ* (-506 to +39) Ap^r^ | This study |
| CP177 | AP379/integrated pLFT*yqjC’-‘lacZ* (-763 to +102) Ap^r^ | This study |
| CP179 | AP379/integrated pLFT*yqjCD’-‘lacZ* (-1142 to +499) Ap^r^ | This study |
| CP181 | AP379/integrated pLFX*bdm-lacZ* (-519 to -1) Ap^r^ | This study |
| CP183 | AP379/integrated pLFX*elaB-lacZ* (-500 to -1) Ap^r^ | This study |
| CP185 | AP379/integrated pLFX*hpf-lacZ* (-641 to -1) Ap^r^ | This study |
| CP187 | AP379/integrated pLFX*raiA-lacZ* (-501 to -1) Ap^r^ | This study |
| CP189 | AP379/integrated pLFX*rmf-lacZ* (-489 to -1) Ap^r^ | This study |
| CP191 | AP379/integrated pLFX*rsd*1*-lacZ* (-497 to -1) Ap^r^ | This study |
| CP193 | AP379/integrated pLFX*rsd2-lacZ* (-497 to +96) Ap^r^ | This study |
| CP195 | AP379/integrated pLFX*sra-lacZ* (-814 to -1) Ap^r^ | This study |
| CP197 | AP379/integrated pLFX*ygaM-lacZ* (-506 to -1) Ap^r^ | This study |
| CP199 | AP379/integrated pLFX*yqjC-lacZ* (-763 to -1) Ap^r^ | This study |
| CP201 | AP379/integrated pLFX*yqjD-lacZ* (-1142 to -1) Ap^r^ | This study |
| CP203 | AP379/integrated placUV5*bdm′-′lacZ* (+1 to +100) Ap^r^ | This study |
| CP205 | AP379/integrated placUV5*elaB′-′lacZ* (+1 to +31) Ap^r^ | This study |
| CP207 | AP379/integrated placUV5*hpf′-′lacZ* (+1 to +277) Ap^r^ | This study |
| CP209 | AP379/integrated placUV5*raiA′-′lacZ* (+1 to +55) Ap^r^ | This study |
| CP211 | AP379/integrated placUV5*rmf′-′lacZ* (+1 to +78) Ap^r^ | This study |
| CP213 | AP379/integrated placUV5*rsd1′-′lacZ* (+1 to +157) Ap^r^ | This study |
| CP215 | AP379/integrated placUV5*rsd2′-′lacZ* (+96 to +157) Ap^r^ |  |
| CP217 | AP379/integrated placUV5*sra′-′lacZ* (+1 to +100) Ap^r^ | This study |
| CP219 | AP379/integrated placUV5*ygaM′-′lacZ* (+1 to +39) Ap^r^ | This study |
| CP221 | AP379/integrated placUV5*yqjC*’*-‘lacZ* (+1 to +102) Ap^r^ | This study |
| CP223 | AP379/integrated placUV5*yqjCD*’*-‘lacZ* (+1 to +499) Ap^r^ | This study |
| CP225 | AP379/integrated pLFT*bdm:sra′-′lacZ* Ap^r^ *csrA::kan* Km^r^ | This study |
| CP227 | AP379/integrated pLFT*elaB′-′lacZ* Ap^r^ *csrA::kan* Km^r^ | This study |
| CP229 | AP379/integrated pLFT*hpf′-′lacZ* Ap^r^ *csrA::kan* Km^r^ | This study |
| CP231 | AP379/integrated pLFT*raiA′-′lacZ* Ap^r^ *csrA::kan* Km^r^ | This study |
| CP233 | AP379/integrated pLFT*rmf′-′lacZ* Ap^r^ *csrA::kan* Km^r^ | This study |
| CP235 | AP379/integrated pLFT*rsd′-′lacZ* Ap^r^ *csrA::kan* Km^r^ | This study |
| CP237 | AP379/integrated pLFT*ygaM’-‘lacZ* Ap^r^ *csrA::kan* Km^r^ | This study |
| CP239 | AP379/integrated pLFT*yqjC’-‘lacZ* Ap^r^ *csrA::kan* Km^r^ | This study |
| CP241 | AP379/integrated pLFT*yqjCD’-‘lacZ* Ap^r^ *csrA::kan* Km^r^ | This study |
| CP243 | AP379/integrated pLFX*bdm-lacZ* Ap^r^ *csrA::kan* Km^r^ | This study |
| CP245 | AP379/integrated pLFX*elaB-lacZ* Ap^r^ *csrA::kan* Km^r^ | This study |
| CP247 | AP379/integrated pLFX*hpf-lacZ* Ap^r^ *csrA::kan* Km^r^ | This study |
| CP249 | AP379/integrated pLFX*raiA-lacZ* Ap^r^ *csrA::kan* Km^r^ | This study |
| CP251 | AP379/integrated pLFX*rmf-lacZ* Ap^r^ *csrA::kan* Km^r^ | This study |
| CP253 | AP379/integrated pLFX*rsd*1*-lacZ* Ap^r^ *csrA::kan* Km^r^ | This study |
| CP255 | AP379/integrated pLFX*rsd2-lacZ* Ap^r^ *csrA::kan* Km^r^ | This study |
| CP257 | AP379/integrated pLFX*sra-lacZ* Ap^r^ *csrA::kan* Km^r^ | This study |
| CP259 | AP379/integrated pLFX*ygaM-lacZ* Ap^r^ *csrA::kan* Km^r^ | This study |
| CP261 | AP379/integrated pLFX*yqjC-lacZ* Ap^r^ *csrA::kan* Km^r^ | This study |
| CP263 | AP379/integrated pLFX*yqjD-lacZ* Ap^r^ *csrA::kan* Km^r^ | This study |
| CP265 | AP379/integrated placUV5*bdm′-′lacZ* Ap^r^ *csrA::kan* Km^r^ | This study |
| CP267 | AP379/integrated placUV5*elaB′-′lacZ* Ap^r^ *csrA::kan* Km^r^ | This study |
| CP269 | AP379/integrated placUV5*hpf′-′lacZ* Ap^r^ *csrA::kan* Km^r^ | This study |
| CP271 | AP379/integrated placUV5*raiA′-′lacZ* Ap^r^ *csrA::kan* Km^r^ | This study |
| CP273 | AP379/integrated placUV5*rmf′-′lacZ* Ap^r^ *csrA::kan* Km^r^ | This study |
| CP275 | AP379/integrated placUV5*rsd′-′lacZ* Ap^r^ *csrA::kan* Km^r^ | This study |
| CP277 | AP379/integrated placUV5*sra′-′lacZ* Ap^r^ *csrA::kan* Km^r^ | This study |
| CP279 | AP379/integrated placUV5*ygaM′-′lacZ* Ap^r^ *csrA::kan* Km^r^ | This study |
| CP281 | AP379/integrated placUV5*yqjC*’*-‘lacZ* Ap^r^ *csrA::kan* Km^r^ | This study |
| CP283 | AP379/integrated placUV5*yqjCD*’*-‘lacZ* Ap^r^ *csrA::kan* Km^r^ | This study |
| CP285 | AP379/integrated placUV5*yqjD*’*-‘lacZ* (short, Fig 11A) Ap^r^ | This study |
| CP287 | AP379/integrated placUV5*yqjD*’*-‘lacZ* Ap^r^ *csrA::kan* Km^r^ | This study |
| CP289 | AP379/integrated pLFT*yqjC’-‘lacZ* Ap^r^ (BS1:AAT to AGT) | This study |
| CP291 | AP379/integrated pLFT*yqjCD’-‘lacZ* Ap^r^ (BS1:AAT to AGT) | This study |
| CP293 | AP379/integrated pLFT*yqjCD’-‘lacZ* Ap^r^ (BS2:GGA to GAA) | This study |
| CP295 | AP379/integrated pLFT*yqjCD’-‘lacZ* Ap^r^ (BS3:GGA to AGA) | This study |
| CP297 | AP379/integrated pLFT*yqjCD’-‘lacZ* Ap^r^ (BS4:CCT to CGT) | This study |
| CP299 | AP379/integrated placUV5-*yqjC*’*-‘lacZ* Ap^r^ (BS1:AAT to AGT) | This study |
| CP301 | AP379/integrated placUV5*yqjD*’*-‘lacZ­* | This study |
| PLB3427 | S17 λpir/pYH411 Ap^r^ | This study |
| PLB3428 | S17 λpir/pYH412 Ap^r^ | This study |
| PLB3434 | S17 λpir/pYH413 Ap^r^ | This study |
| CF7789 | F^−^ λ^−^Δ*lacI-lacZ* (*Mlu*I) | (3) |
| PLB982 | CF7789 *Δrpos::tet* | (4) |
| *Plasmids* |  |  |
| pLFT | Used for constructing translational fusions; Ap^r^ | (5) |
| pPFINT | Helper plasmid used for integrating *lacZ* fusions into the chromosome; Tc^r^ | (5) |
| pBR322 | Cloning vector; Tc^r^ Ap^r^ | (6) |
| pCEP125 | pLFT*bdm:sra′-′lacZ* Ap^r^ | This study |
| pCEP126 | pLFT*elaB′-′lacZ* Ap^r^ | This study |
| pCEP127 | pLFT*hpf′-′lacZ* Ap^r^ | This study |
| pCEP128 | pLFT*raiA′-′lacZ* Ap^r^ | This study |
| pCEP129 | pLFT*rmf′-′lacZ* Ap^r^ | This study |
| pCEP130 | pLFT*rsd′-′lacZ* Ap^r^ | This study |
| pCEP131 | pLFT*ygaM’-‘lacZ* Ap^r^ | This study |
| pCEP132 | pLFT*yqjC’-‘lacZ* Ap^r^ | This study |
| pCEP133 | pLFT*yqjCD’-‘lacZ* Ap^r^ | This study |
| pCEP134 | pLFX*bdm-lacZ* Ap^r^ | This study |
| pCEP135 | pLFX*elaB-lacZ* Ap^r^ | This study |
| pCEP136 | pLFX*hpf-lacZ* Ap^r^ | This study |
| pCEP137 | pLFX*raiA-lacZ* Ap^r^ | This study |
| pCEP138 | pLFX*rmf-lacZ* Ap^r^ | This study |
| pCEP139 | pLFX*rsd*1*-lacZ* Ap^r^ | This study |
| pCEP140 | pLFX*rsd2-lacZ* Ap^r^ | This study |
| pCEP141 | pLFX*sra-lacZ* Ap^r^ | This study |
| pCEP142 | pLFX*ygaM-lacZ* Ap^r^ | This study |
| pCEP143 | pLFX*yqjC-lacZ* Ap^r^ | This study |
| pCEP144 | pLFX*yqjD-lacZ* Ap^r^ | This study |
| pCEP145 | placUV5*bdm′-′lacZ* Ap^r^ | This study |
| pCEP146 | placUV5*elaB′-′lacZ* Ap^r^ | This study |
| pCEP147 | placUV5*hpf′-′lacZ* Ap^r^ | This study |
| pCEP148 | placUV5*raiA′-′lacZ* Ap^r^ | This study |
| pCEP149 | placUV5*rmf′-′lacZ* Ap^r^ | This study |
| pCEP150 | placUV5*rsd′-′lacZ* Ap^r^ | This study |
| pCEP151 | placUV5*sra′-′lacZ* Ap^r^ | This study |
| pCEP152 | placUV5*ygaM′-′lacZ* Ap^r^ | This study |
| pCEP153 | placUV5*yqjC*’*-‘lacZ* Ap^r^ | This study |
| pCEP154 | placUV5*yqjCD*’*-‘lacZ* Ap^r^ | This study |
| pCEP155 | pLFT*yqjC’-‘lacZ* Ap^r^ (BS1:AAT to AGT) | This study |
| pCEP156 | pLFT*yqjCD’-‘lacZ* Ap^r^ (BS1:AAT to AGT) | This study |
| pCEP157 | pLFT*yqjCD’-‘lacZ* Ap^r^ (BS2:GGA to GAA) | This study |
| pCEP158 | pLFT*yqjCD’-‘lacZ* Ap^r^ (BS3:GGA to AGA) | This study |
| pCEP159 | pLFT*yqjCD’-‘lacZ* Ap^r^ (BS4:CCT to CGT) | This study |
| pCEP160 | placUV5-*yqjC*’*-‘lacZ* Ap^r^ (BS1:AAT to AGT) | This study |
| pCEP161 | placUV5*yqjCD*’*-‘lacZ* Ap^r^ | This study |
| pCEP162 | placUV5*yqjD*’*-‘lacZ* Ap^r^ | This study |
| pCEP163 | placUV5*yqjD*’*-‘lacZ* (short, Fig 11A) Ap^r^ | This study |
| pYH411 | P_T7_- *yqjC’-‘lacZ* from nt -82 to +5 relative to the *yqjC* TLN start codon into pLFT Ap^r^ | This study |
| pYH412 | P_T7_- *yqjD’-‘lacZ* from nt -108 to +11 relative to the *yqjD* TLN start codon into pLFT Ap^r^ | This study |
| pYH413 | P_T7_- *elaB’-‘lacZ* from nt -26 to +2 relative to the *elaB* TXN start codon into pLFT Ap^r^ | This study |
| *Bacteriophage* |  |  |
| P1vir | Strictly lytic P1 | Carol Gross |

^a^Numbers in parentheses indicate the cloned region relative to the furthest 5’ start of transcription (unless otherwise indicated), as well as *yqjCD* and *yqjD* mutations

^b^Fusions were integrated into the *E. coli* λ *att* site

**References**

1. Pannuri A, Vakulskas CA, Zere T, McGibbon LC, Edwards AN, Georgellis D, Babitzke P, Romeo T. 2016. Circuitry linking the catabolite repression and Csr global regulatory systems of Escherichia coli. J Bacteriol 198:3000–3015.

2. Romeo T, Gong M, Liu MY, Brun-Zinkernagel AM. 1993. Identification and molecular characterization of csrA, a pleiotropic gene from Escherichia coli that affects glycogen biosynthesis, gluconeogenesis, cell size, and surface properties. J Bacteriol 175:4744–4755.

3. Wei BL, Brun-Zinkernagel AM, Simecka JW, Prüss BM, Babitzke P, Romeo T. 2001. Positive regulation of motility and flhDC expression by the RNA-binding protein CsrA of Escherichia coli. Mol Microbiol 40:245–256.

4. Costanzo A, Ades SE. 2006. Growth phase-dependent regulation of the extracytoplasmic stress factor, σ^E^, by guanosine 3’,5’-bispyrophosphate (ppGpp). J Bacteriol 188:4627–4634.

5. Edwards AN, Patterson-Fortin LM, Vakulskas CA, Mercante JW, Potrykus K, Vinella D, Camacho MI, Fields JA, Thompson SA, Georgellis D, Cashel M, Babitzke P, Romeo T. 2011. Circuitry linking the Csr and stringent response global regulatory systems. Mol Microbiol 80:1561–1580.

6. Sircili MP, Walters M, Trabulsi LR, Sperandio V. 2004. Modulation of enteropathogenic Escherichia coli virulence by quorum sensing. Infect Immun 72:2329–2337.
