# Supplementary material for: CsrA coordinates the expression of ribosome hibernation and anti-σ factor proteins
Source: mBio. 2023 Nov 9;14(6):e02585-23. doi: 10.1128/mbio.02585-23 (PMC10746276; doi:10.1128/mbio.02585-23)
Supplement: Table S2 — Primers used in this study. [file mbio.02585-23-s0005.docx]

Table S2. Primers used in this study

| Oligonucleotide^a^ | Sequence | Purpose |
| --- | --- | --- |
| bdm-sra’-‘lacZ fwd | 5' GAGGAGCTGCAGCAATCGATGTTGGCGTGAATCC 3' | Forward primer for constructing translational fusion |
| bdm-sra’-‘lacZ rev | 5' CTCCTCGGATCCTGACGGTTCGATTTCATTTTAACTCC 3' | Reverse primer for constructing translational fusion |
| elaB’-‘lacZ fwd | 5' GAGGAGCTGCAGGGAGAGCATTATGATTGAATGGC 3' | Forward primer for constructing translational fusion |
| elaB’-‘lacZ rev | 5' CTCCTCGGATCCGACATTCTCGTTCTCCATTTGC 3' | Reverse primer for constructing translational fusion |
| raiA’-‘lacZ fwd | 5' GAGGAGCTGCAGCTATCCGAACAGTCAGTACACC 3' | Forward primer for constructing translational fusion |
| raiA’-‘lacZ rev | 5' CTCCTCGGATCCGTCATAAATTTTACCTCTTGTCTTCCC 3' | Reverse primer for constructing translational fusion |
| rmf’-‘lacZ fwd | 5’ GAGGAGCTGCAGGTTGCCAACCTGAGTACG 3’ | Forward primer for constructing translational fusion |
| rmf’-‘lacZ rev | 5' CTCCTCGGATCCTCTCGTTTTTGTCTCTTCATGC 3' | Reverse primer for constructing translational fusion |
| rsd’-‘lacZ fwd | 5' GAGGAGCTGCAG ACTTTAATTCCGCTCTCTTCC 3' | Forward primer for constructing translational fusion |
| rsd’-‘lacZ rev | 5' CTCCTCGGATCCGGTCGGTTAAACATATGATCCC 3' | Reverse primer for constructing translational fusion |
| rmf’-‘lacZ fwd | 5’ GAGGAGCTGCAGGTTGCCAACCTGAGTACG 3’ | Forward primer for constructing translational fusion |
| rmf’-‘lacZ rev | 5' CTCCTCGGATCCTCTCGTTTTTGTCTCTTCATGC 3' | Reverse primer for constructing translational fusion |
| ygaM’-‘lacZ fwd | 5' GAGGAGCTGCAGCTTCTTTTCGCGACTGGC 3' | Forward primer for constructing translational fusion |
| ygaM’-‘lacZ rev | 5' CTCCTCGGATCCGGTCGGTTAAACATATGATCCC 3' | Reverse primer for constructing translational fusion |
| yqjC’-‘lacZ fwd | 5' GAGGAGCTGCAGTTCATAAACACGGTTTATCGGC 3' | Forward primer for constructing translational fusion |
| yqjC’-‘lacZ rev | 5' CTCCTCGGATCCAAAGCGATGCGGTATTTCATTCTTG 3' | Reverse primer for constructing translational fusion |
| yqjCD’-‘lacZ fwd | 5' GAGGAGCTGCAGTTCATAAACACGGTTTATCGGC 3' | Forward primer for constructing translational fusion |
| yqjCD’-‘lacZ rev | 5' CTCCTCGGATCCTCTTTCGACATAGTTTTCTCCAGG 3' | Reverse primer for constructing translational fusion |
| bdm-lacZ rev | 5' CTCCTCGGATCCACATTCAGCATGGCAAATATTTG 3' | Reverse primer for constructing transcriptional fusion |
| elaB-lacZ fwd | 5' GAGGAGCTGCAGGCAAGATCTGCATCACTCTG 3' | Forward primer for constructing transcriptional fusion |
| elaB-lacZ rev | 5' CTCCTCGGATCCTGTCACTAACTATAGACAAGGGTTG 3' | Reverse primer for constructing transcriptional fusion |
| raiA-lacZ rev | 5' CTCCTCGGATCCGATGTCAAAATGTGTGATGAAAATCTC 3' | Reverse primer for constructing transcriptional fusion |
| rmf-lacZ rev | 5' CTCCTCGGATCCGCAATCACAGTTAAGCAATACCG 3' | Reverse primer for constructing transcriptional fusion |
| rsd1-lacZ rev | 5' CTCCTCGGATCCGAACAAAAATTATGGTTGCCGAAGG 3' | Reverse primer for constructing transcriptional P1 fusion |
| rsd2-lacZ fwd | 5' GAGGAGCTGCAGATGGCTGACGACCC 3' | Forward primer for constructing transcriptional P2 fusion |
| rsd2-lacZ rev | 5' CTCCTCGGATCCAACCATGTTAAACATGCCAGTG 3' | Reverse primer for constructing transcriptional P2 fusion |
| sra-lacZ rev | 5' CTCCTCGGATCCTGTCACTAACTATAGACAAGGGTTG 3' | Reverse primer for constructing transcriptional fusion |
| ygaM-lacZ rev | 5' CTCCTCGGATCCCGATGTAATTTTCAGTGTAGACC 3' | Reverse primer for constructing transcriptional fusion |
| elaB’-‘lacZ fwd pUV5 | 5’ GAGGAGGAATTCAGGTTTTACGCAAATGGAGAACGAGAAT  GTCGGATCCGAGGAG 3’ | Oligo for annealing to construct leader fusion |
| elaB’-‘lacZ rev pUV5 | 5’ CTCCTCGGATCCGACATTCTCGTTCTCCATTTGCGTAAAA  CCTGAATTCCTCCTC 3’ | Oligo for annealing to construct leader fusion |
| yqjC-lacZ fwd pUV5 | 5' GAGGAGGAATTCTAGGTCCGAATCACAATGGAAGGTTCA  AGAATGAAATACCGCATCGCTTTGGATCCGAGGAG 3' | Oligo for annealing to construct leader fusion |
| yqjC-lacZ rev pUV5 | 5' CTCCTCGGATCCAAAGCGATGCGGTATTTCATTCTTGAAC  CTTCCATTGTGATTCGGACCTAGAATTCCTCCTC 3' | Oligo for annealing to construct leader fusion |
| yqjC-lacZ fwd pUV5 | 5' GAGGAGGAATTCTAGGTCCGAATCACAATGGAAGGTTCA  AGAATGAAATACCGCATCGCTTTGGATCCGAGGAG 3' | Oligo for annealing to construct leader fusion |
| yqjC-lacZ rev pUV5 | 5' CTCCTCGGATCCAAAGCGATGCGGTATTTCATTCTTGAAC  CTTCCATTGTGATTCGGACCTAGAATTCCTCCTC 3' | Reverse primer for constructing leader fusion |
| yqjD-lacZ fwd pUV5 full | 5' GAGGAGGAATTCTGCCAAACGCGAACG 3' | Fwd primer for constructing full leader fusion |
| yqjD-lacZ fwd pUV5 short | 5' GAGGAGGAATTCCTCACAATAGTCACTACTTACTCACCTG  GAGAAAACTATGTCGAAAGAGGATCCGAGGAG 3' | Oligo for annealing to construct leader fusion |
| yqjD-lacZ rev pUV5 short | 5' CTCCTCGGATCCTCTTTCGACATAGTTTTCTCCAGGTGAG  TAAGTAGTGACTATTGTGAGGAATTCCTCCTC 3' | Oligo for annealing to construct leader fusion |
| elaB T7 native | 5' TAATACGACTCACTATAGGGTTTTACGCAAATGGAGAACG  AGAATGTCTAATCAG 3' | Oligo for annealing for in vitro transcription |
| elaB T7 native complement | 5' CTGATTAGACATTCTCGTTCTCCATTTGCGTAAAACCCTAT  AGTGAGTCGTATTA 3' | Oligo for annealing for in vitro transcription |
| elaB T7 CCA | 5' TAATACGACTCACTATAGGGTTTTACGCAAATCCAGAACG  AGAATGTCTAATCAG 3' | Oligo for annealing for in vitro transcription |
| elaB T7 CCA complement | 5' CTGATTAGACATTCTCGTTCTGGATTTGCGTAAAACCCTAT  AGTGAGTCGTATTA 3' | Oligo for annealing for in vitro transcription |
| yqjD T7 native | 5' TAATACGACTCACTATAGGGCGGTGGTTAACACTACCGCT  TCGTTGTCTGGACAATCGTTCCTTTGTAATAGGTCCGAATCACAATGGAAGGTTCAAGAATGAAATACCGCATCGCTTTAGCTGTT 3' | gBlock for in vitro transcription |
| yqjC T7 GGA1 mut | 5' TAATACGACTCACTATAGGGCGGTGGTTAACACTACCGCT  TCGTTGTCTAAACAATCGTTCCTTTGTAATAGGTCCGAATCACAATGGAAGGTTCAAGAATGAAATACCGCATCGCTTTAGCTGTT 3' | gBlock for in vitro transcription |
| yqjC T7 GGA2 mut | 5' TAATACGACTCACTATAGGGCGGTGGTTAACACTACCGCT  TCGTTGTCTGGACAATCGTTCCTTTGTAATAGGTCCGAATCACAATAAAAGGTTCAAGAATGAAATACCGCATCGCTTTAGCTGTT 3' | gBlock for in vitro transcription |
| *elaB* T7 for PstI | 5’ TGCCTGCAGCTAATACGACTCACTATAGGGTTT TACGCAAATG GAGAACGAGA ATG 3’ | PURExpress fusion |
| *elaB* Rev BamHI | 5’ GCGGGATCCGACATTCTCGTTCTCCATTTGCGTAAAACC3’ | PURExpress fusion |
| *yqjC* T7 for PstI | 5’ GGCCTGCAGCTAATACGACTCACTATAGGGAGGCGGTGG  TTAACACTACCGC 3’ | PURExpress fusion |
| *yqjD* T7 for PstI | 5’ GGCCTGCAGCTAATACGACTCACTATAGGGTTGCCAAAC  GCGAACGGAAACTGGC 3’ | PURExpress fusion |
| *yqjC* rev BamHI | 5’ GGTGGATCCTTCATTCTTGAACCTTCCATTGTGATTCGG 3’ | PURExpress fusion |
| *yqjD* rev BamHI | 5’ CGCGGATCCTCTTTCGACATAGTTTTCTCCAGGTGAG 3’ | PURExpress fusion, footprint |
| *yqjC* Rev | 5’GAGCAAAGAGAGAAACAGCTAAAGCGATGC 3’ | footprint, toeprint |
| *LacZ* | 5’ GCAAGGCGATTAAGTTGGG 3’ | toeprint *yqjD* |
| *yqjD* PE1 | 5’ CTTTCGACATAGTTTTCTCCAGG 3’ | Primer extension of *yqjD* |
| *yqjC* PE 23 | 5’ GGGTAGTGGCATAACTACC 3’ | Primer extension of *yqjD,* in vitro transcription |
| TXN For | 5’ CCTCGACAGAACATTGCCCCATG 3’ | in vitro transcription |
| yqjC/CD A16G | 5' TCCTTTGTAATAGGTCCGAATCACAGTGGAAGGTTCAAG 3' | Primer for site directed mutagenesis |
| yqjC/CD A16G complement | 5' CTTGAACCTTCCACTGTGATTCGGACCTATTACAAAGGA 3' | Primer for site directed mutagenesis |
| yqjD GGA1 | 5' ATTGCCAAACGCGAACAGAAACTGGCAGAAGCG 3' | Primer for site directed mutagenesis |
| yqjD GGA1 complement | 5' CGCTTCTGCCAGTTTCTGTTCGCGTTTGGCAAT 3' | Primer for site directed mutagenesis |
| yqjD GGA2 | 5' AACTGGCAGAAGCGCAGAAAGAGCTGAAAAAGCTG 3' | Primer for site directed mutagenesis |
| yqjD GGA complement | 5' CAGCTTTTTCAGCTCTTTCTGCGCTTCTGCCAGTT 3' | Primer for site directed mutagenesis |
| yqjD GGA3 | 5' GGAAGAGCTGAAAAAGCTAGAAGCGCGCGACTA 3' | Primer for site directed mutagenesis |
| yqjD GGA3 complement | 5' TAGTCGCGCGCTTCTAGCTTTTTCAGCTCTTCC 3' | Primer for site directed mutagenesis |
| yqjD GGA4 | 5'CAATAGTCACTACTTACTCACGTGGAGAAAACTATGTCGAAAG 3' | Primer for site directed mutagenesis |
| yqjD GGA4 complement | 5'CTTTCGACATAGTTTTCTCCACGTGAGTAAGTAGTGACTATTG 3' | Primer for site directed mutagenesis |

^a^Primers and gBlocks were purchased from Integrated DNA Technologies
